# Chemical Profiles and Identification of Key Compound Caffeine in Marine-Derived Traditional Chinese Medicine *Ostreae concha*

**DOI:** 10.3390/md10051180

**Published:** 2012-05-23

**Authors:** Xue Yang, Shi-Lu Zhou, Ai-Cui Ma, Hai-Tao Xu, Hua-Shi Guan, Hong-Bing Liu

**Affiliations:** 1 Key Laboratory of Marine Drugs, Chinese Ministry of Education, School of Medicine and Pharmacy, Ocean University of China, Qingdao 266003, China; Email: yangxue95@163.com (X.Y.); maaicui8718@sina.com (A.-C.M.); 2 Technology Center of China Tobacco Shandong Industrial Corporation, Qingdao 266101, China; Email: zhoushilu2004@126.com (S.-L.Z.); xuhaitaodahai@163.com (H.-T.X.)

**Keywords:** oyster shell, traditional Chinese medicine, chemical profile, principal component analysis, caffeine

## Abstract

To compare the chemical differences between the medicinal and cultured oyster shells, their chemical profiles were investigated. Using the ultra performance liquid chromatography-electron spraying ionization-mass spectrometry (UPLC-ESI-MS), combined with principal component analysis (PCA) and orthogonal projection to latent structures discriminant analysis (OPLS-DA), the discrimination of the chemical characteristics among the medicinal and cultured oyster shells was established. Moreover, the chemometric analysis revealed some potential key compounds. After a large-scale extraction and isolation, one target key compound was unambiguously identified as caffeine (**1**) based on extensive spectroscopic data analysis (1D and 2D NMR, MS, and UV) and comparison with literature data.

## 1. Introduction

The oyster is a traditional and popular seafood in most coastal countries and its cultivation has become an industry. Over the past twenty years, oyster cultivation has greatly developed in China. Oysters are the largest commercial molluscan group cultured in China, even in the world. According to the statistical data published by the food and agriculture organization of the United Nations (FAO), the production of oysters reached 350 metric tons in 2009 in China, accounting for 81.4% of total oyster production in the world [1]. The oyster meat is eaten, however the shells of oysters were mostly discarded. Enormous “oyster shell-mountains” can be seen in the oyster culture areas and this has become a big environmental issue. The disposal or application of the remaining shells is therefore investigated from many aspects. Actually, *Ostreae concha* (Pinyin: Mu-li), the shell of oysters has been widely used in traditional Chinese medicine (TCM). It was first documented in Shen Nong Ben Cao Jing and first used in prescriptions by Zhong-jing Zhang (150–219 AD). *Ostreae concha* is commonly used with other herbs in prescriptions to treat symptoms such as palpitations, insomnia, dizziness, tinnitus, scrofula, subcutaneous nodules, and abdominal mass. Pharmacological effects of *Ostreae concha* include strengthening the immune system, anti-gastric ulcer, sedation, anti-tumor, anti-virus, *etc.* [2,3].

The shells of three species of oyster [*Crassostrea gigas* Thunberg (*Ostrea gigas* Thunberg), *Crassostrea talienwhanensis* Crosse and *Crassostrea rivularis* Gould] are together listed in the 2010 Edition of the Chinese Pharmacopoeia (CP). The process of harvesting the medicinal materials of *Ostreae concha* involves the collection of oyster shells over the whole year, removal of the meat and drying the clean shell in the sun. Determination of calcium carbonate content is the only quality control measure for *Ostreae concha* [4]. There are no requirements for declaration of the natural source or cultured source, nor of the nuber of years of growth oysters in the CP. According to our investigation, natural perennial *C. gigas* is a main source for medicinal materials, while shells of edible oysters that were cultured for one to three years are seldom used in the clinic. It is still unknown whether the cultured oyster shells possess potential medicinal values or if they can replace the natural perennial ones in TCM. As different chemical compositions result in different pharmacological effects, comprehensive quality control methods for *Ostreae concha* should be established. In view of this situation, it is of urgent importance to clarify the active ingredients of TCM *Ostreae concha* and compare the ingredients between the medicinal shells and the cultured shells.

Calcium carbonate has been confirmed to be the major effective ingredient in *Ostreae concha* [5,6]. Its weight proportion should be above 94.0% according to the CP. However, calcium carbonate is also the most abundant component (≥95.0%) in some other marine-shell TCMs, such as *Haliotidis Concha* (Pinyin: Shi-Jue-Ming), *Arcae concha* (Pinyin: Wa-Leng-Zi), *Meretricis concha* (Pinyin: Ge-Ke), *Cyclinae concha* (Pinyin: Ge-Ke) [4]. Since the function and usage of these other marine shells are different from *Ostreae concha*, we therefore hypothesize that trace organic components and/or minor inorganic elements are also potential bioactive ingredients. In our research for new biologically active constituents from marine-shell TCM, *Ostreae concha* attracted our attention as its crude methanol extract showed good cytotoxic activity against the human hepatoma cell line BEL-7402, cervical cancer cell line HeLa and murine leukemic cell line P388 [7].

Until now, only few documents focused on the organic matrix of oyster shells [8]. The analysis of organic components of *Ostreae concha* remains a difficult task due to its low concentrations and high complexity. To address this problem, the ultra performance liquid chromatography-mass spectrometry (UPLC-MS) is utilized here as it is a powerful tool for analyzing complex mixtures. A typical UPLC-MS chromatogram of a complex chemical mixture contains a large amount of chemical information, provided in a relatively short time [9]. The objectives of this study were to (1) study the chemical profiles of oyster shells from different sources applying the UPLC-MS method; (2) clarify the potential key compounds differing between various sources of oyster shells based on principal component analysis (PCA) and orthogonal projection to latent structures discriminant analysis (OPLS-DA); (3) carry out a preliminary evaluation of the potential medicinal value of cultured oyster shells.

## 2. Results and Discussion

### 2.1. Chemical Profiles Analysis

**Figure 1:**
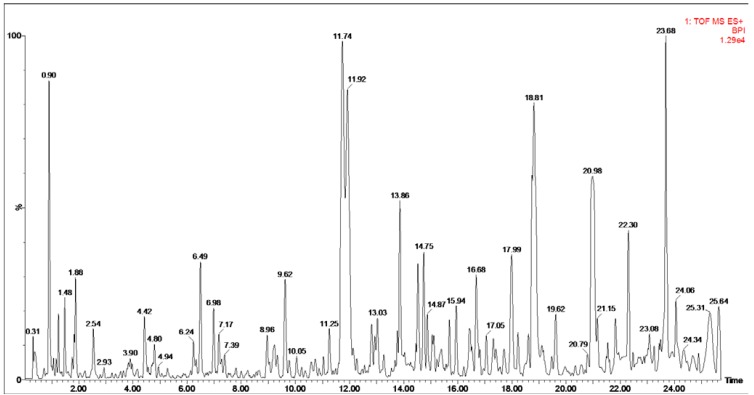
Base peak intensity (BPI) chromatogram of sample A11 in positive ion mode. Column: Waters ACQUITYTM UPLC BEH C18 column (50 mm×2.1 mm i.d., 1.7 μm). Flow rate: 0.4 mL·min^−1^. Column temperature: 35°C. Mobile phase: a linear gradient elution with 0.1% formic acid in water and 0.1% formic acid in acetonitrile. Ion source: electrospray interface (ESI).

Twenty-four oyster shell samples purchased from different drugstores, aquatic product markets and farms were obtained and prepared for UPLC-MS analysis (Table 1). Both positive and negative ion modes of electrospray interface (ESI) were used in UPLC-MS analysis to result in exhaustive information. After optimization of chromatographic parameters, 459 to 562 masses in positive ion mode and 432 to 595 masses in negative ion mode were recorded in base peak intensity (BPI) chromatograms of each oyster shell. According to the extracted mass-retention time pairs (EMRT) and the intensity of peaks in the BPI chromatograms (Figure 1,others are provided as supplementary files), the 24 shell samples were dissimilar due to the chemical diversity.

**Table 1:** Oyster shellsamples.

No.	Source	Time	Site	Growth Years	Species
A1	Medicinal materials *^a^*	August, 2008	Anguo, Hebei	unknown *^ b^*	*^c^*
A2		December, 2009	Bozhou, Anhui	perennial	
A3		September, 2010	Bozhou, Anhui	unknown *^b^*	
A4		May, 2010	Yishui, Shandong	perennial	
A5		October, 2010	Heze, Shandong	perennial	
A6		August, 2009	Kaifeng, Henan	unknown^*b*^	
A7		February, 2010	Qingdao, Shandong	unknown *^b^*	
A8		September,2009	Weifang, Shandong	perennial	
A9		October, 2010	Beijing	unknown *^b^*	
A10		June, 2010	Wuhan, Hubei	perennial	
A11		May, 2010	Anguo, Hebei	perennial	
A12		October, 2011	Anguo, Hebei	perennial	
B1	Cultured materials *^d^*	October, 2009	Rongcheng, Shandong	2	*C. gigas*
B2		October, 2008	Rongcheng, Shandong	1	*C. gigas*
B3		October, 2009	Qingdao, Shandong	1	*C. gigas*
B4		October, 2009	Yantai, Shandong	1	*C. gigas*
B5		October, 2009	Rushan, Shandong	1	*C. gigas*
B6		October, 2009	Lianyungang, Jiangsu	2	*C. gigas*
B7		October, 2010	Rongcheng, Shandong	2	*C. talienwhanensis*
B8		October, 2009	Rongcheng, Shandong	3	*C. talienwhanensis*
B9		June, 2010	Zhanjiang, Guangdong	2	*C. rivularis*
B10		June, 2010	Lianyungang, Jiangsu	3	*C.plicatula*
B11		October, 2008	Rongcheng, Shandong	1.5	*C. gigas*
B12		October, 2009	Rongcheng, Shandong	1.5	*C. talienwhanensis*

*^a^* Purchased from drugstores; *^b^* Obtained as crude decoction pieces; *^c^* Identification not reliable; *^d^* Purchased from aquatic product markets and farms.

**Figure 2:**
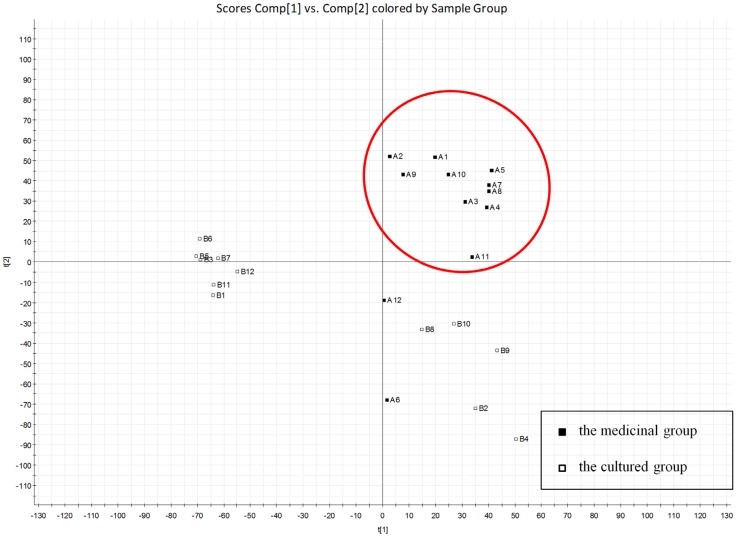
Principal component analysis (PCA) score plot of oyster shell samples in positive ion mode; for their sources refer to Table 1.

To facilitate the identification of differences or similarities among the samples, PCA was used for analyzing the chromatographic data. PCA can discriminate between sample categories by reducing the dimensions of the variables in order to simplify subsequent analysis [10]. Twenty-four samples were separated into blocks in the PCA score plots: samples A1-5, A7-11 were one group, while two different clusters of the cultured materials can be seen in the scatter plot of the positive ion mode (Figure 2). It was interesting that samples A1-5, A7-11 were medicinal shells purchased from drugstores, and samples B1-12 were cultured shells grown for 1 to 3 years. Therefore, PCA allows a clear discrimination of the chemical characteristics among perennial medicinal material shells and cultured shells based on UPLC-MS data. The scattered distribution of the cultured samples revealed that chemical differences existed within the same species even from the same geographic provenience. Ecological factors, such as number of years of growth, local environment and food chain, might contribute to the different metabolites. In addition, oyster shell is a kind of constructive material which could absorb the organic matter in sea water during its growth process. All of these elements might result in the differences of the chemical constituents observed. The deviation of samples A6 and A12 from the main trajectory in Figure 2 indicated that they might not be the perennial shells. Moreover, the results suggested that potential key compounds existed in these two groups of shells, which might be potential efficient substances and could be regarded as determination standards for the quality control of *Ostreae concha*.

### 2.2. Clarification of Potential Key Compounds

**Figure 3:**
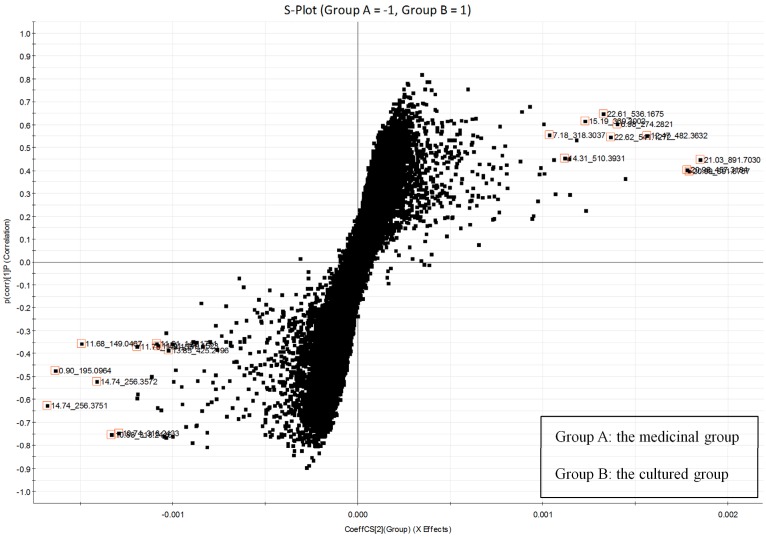
Orthogonal projection to latent structures discriminant analysis (OPLS-DA) S-plot of oyster shell samples in positive ion mode (group A: the medicinal group; group B: the cultured group). Each point represents an individual extracted mass-retention time pair (EMRT). The *Y*-axis denotes confidence of a marker’s contribution to the group differences, and the *X*-axis denotes the contribution of a particular marker to the group differences. The top 20 VIP variables were marked in the S-plots.

OPLS-DA was applied to the chemical profiles in order to better discriminate between perennial medicinal shells and cultured shells [11]. OPLS-DA score plots in the component P1 direction separated oyster shell samples into two blocks, and component P2 properly explained the individual variation in each group. The ions that sit at both ends of the S-plot contributed significantly to the clustering of the two groups and might be regarded as the potential key compounds (Figure 3). Furthermore, all the detected ions were arranged in descending order according to the variant weight parameters (VIP). The top twenty VIP variables were selected as potential key compounds (Table 2).

**Table 2:** The top 20variant weight parameters (VIP)variables in positive ion mode.

No.	Retention Time (min)	Mass (*m/z*)	VIP
1	14.74	256.3751	16.22
2	12.47	482.3632	15.23
3	6.98	274.2821	13.12
4	14.74	256.3572	13.08
5	10.88	318.2416	12.49
6	11.73	149.1551	12.12
7	10.74	318.2433	12.03
8	21.03	891.703	11.86
9	15.19	369.3002	11.48
10	11.68	149.0467	11.41
11	11.91	149.1791	11.40
12	11.91	149.1523	11.20
13	22.61	536.1675	11.06
14	14.31	510.3931	11.02
15	20.99	891.6757	10.99
16	20.96	457.3184	10.98
17	0.90	195.0964	10.97
18	22.62	541.1272	10.83
19	13.85	425.2196	10.21
20	7.18	318.3037	10.17

**Figure 4:**
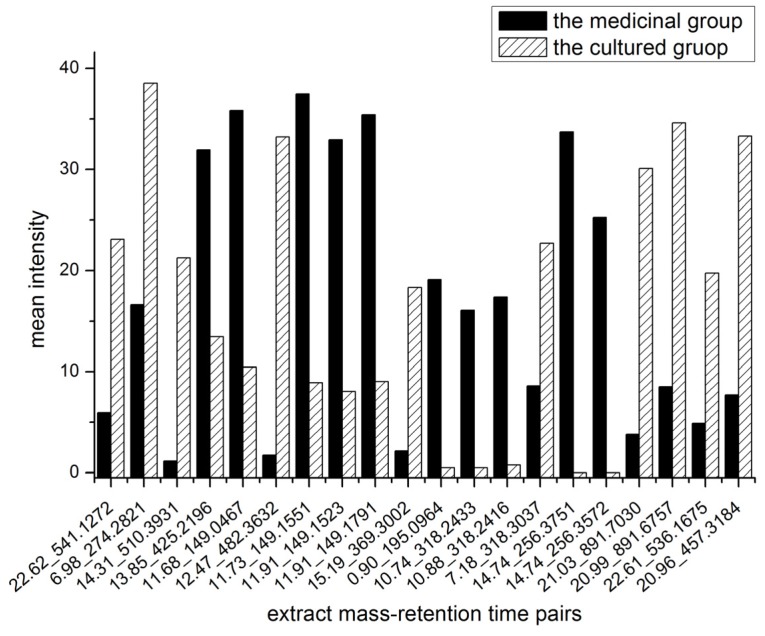
Relative contents of the potential key compounds in medicinal shells and cultured shells in positive ion mode.

**Figure 5:**
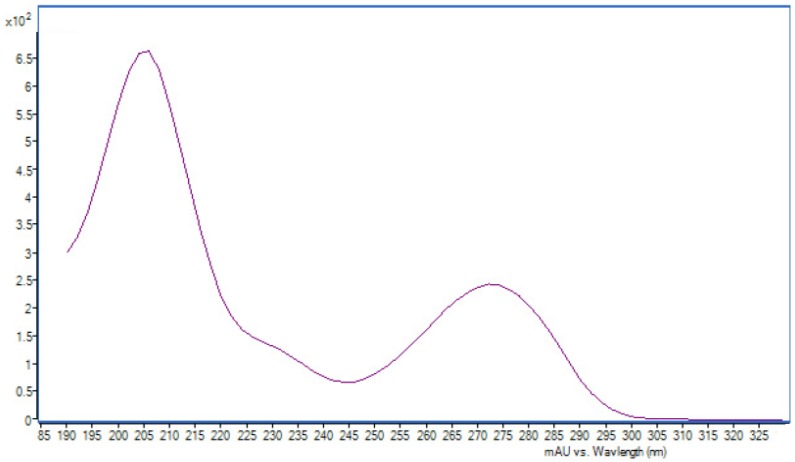
The ultraviolet spectrum of compound **1**.

It is difficult to identify all the key compounds due to the very limited database on the chemical constituents of oyster shells. Natural product isolation is still an inefficient strategy to solve this problem, since the challenge of the low content of the chemical constituents is hard to overcome. After a detailed comparison of the mean peak areas of the potential key compounds (Figure 4), five EMRT pairs that existed mainly in perennial medicinal shells were found. They were 0.90_195.0964, 10.74_318.2433, 10.88_318.2416, 14.74_256.3572 and 14.74_256.3751 in positive mode. With comprehensive analyses of the UPLC-DAD chromatograms, the compound (0.90_195.0964) with UV absorption at 205, 274 nm (Figure 5) was suggested to be separable.

### 2.3. Isolation and Identification of Caffeine

The target compound (0.90_195.0964, **1**) was obtained through repeated chromatographic methods including semi-preparative HPLC from perennial medicinal material of oyster shells.

**Figure 6:**
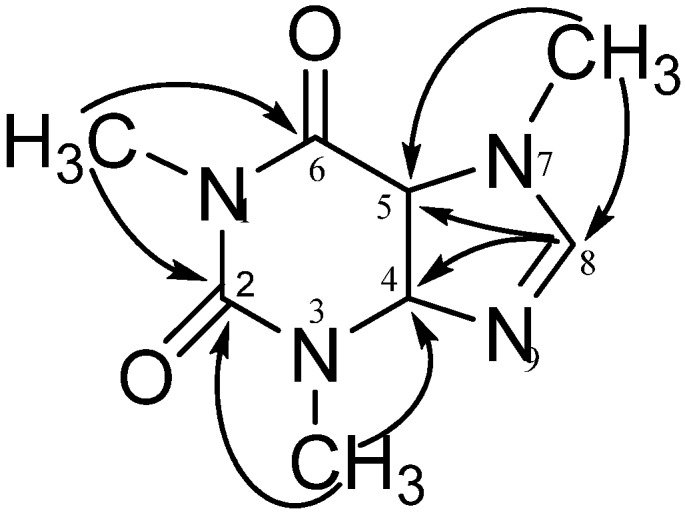
Key HMBC correlations of compound **1**.

Compound **1** was isolated as white needles. The molecular formula C_8_H_10_N_4_O_2_ was determined from positive HR-ESI-MS (*m/z* 195.0882 [M + H]^+^, calc. 195.0886) in association with NMR data. The ^1^H NMR spectrum in DMSO-*d*_6_ exhibited one proton at δ_H_ 8.00 (*s*, H-8), three methyl singles at δ_H_ 3.87 (*s*, N7-CH_3_), 3.40 (*s*, N3-CH_3_) and 3.21 (*s*, N1-CH_3_). The ^13^C NMR spectrum displayed in total 8 resonances, including four carbons in 140 to 155 ppm [C-6 (δ_C_ 154.6), C-2 (δ_C_ 151.1), C-4 (δ_C_ 148.1) and C-8 (δ_C_ 142.8)], and one carbon at δ_C_ 106.6 (C-5), in addition to three methyls at δ_C_ 33.1 (N7-CH_3_), 29.4 (N3-CH_3_) and 27.5 (N1-CH_3_). Based on 2D NMR data analysis (Figure 6) and by comparison with literature data [12,13], compound **1** was identified as caffeine (all spectra are provided as supplementary materials).

It is well known that caffeine is a plant-derived secondary metabolite and is found in coffee plants, tea trees, cocoa trees, as well as in the leaves and fruits of other plants [14,15]. Moreover, caffeine is a stimulant of the central nervous system. It is able to promote secretion of gastric acid and alleviate migraine, and plays wide-ranging roles in other systems of the body [14,16,17,18]. Our findings represent the first report of caffeine found in marine shells. Until now there is only one report that caffeine was found in marine animals and microorganisms: It was isolated from gorgonian by several research groups [19,20,21,22,23]. However, the biological origin of caffeine should be investigated. There is also a need for thorough evaluation of the action of caffeine on TCM *Ostreae concha*.

## 3. Experimental Section

### 3.1. General Experimental Procedures

The UPLC-MS analysis was carried out using a Waters ACQUITY™ ultra performance liquid chromatography system (Waters Corp., Milford, MA, USA) coupled with a Synapt™ Q-TOF High Definition Mass Spectrometry (Waters Corp., Milford, MA, USA). Chromatographic separations were achieved on an ACQUITY™ UPLC BEH C_18_ column (50 mm × 2.1 mm i.d., 1.7 μm; Waters Corp., Milford, MA, USA).

1D and 2D NMR spectra were recorded on the JEOL JNM-ECP 600 spectrometers using TMS as internal standard and chemical shifts were recorded as δ-values. ESI-MS analysis was measured on a Waters Q-TOF ULTIMA GLOBAL GAA076LC mass spectrometer. HPLC analysis was performed by using an Aglient 1100 HPLC system coupled to a photodiode array detector. Routine detection was at 210, 230, 254 and 280 nm. The separation column (250 × 4.6 mm i.d., 5 μm) was prefilled with YMC C_18_, and a linear gradient of 0.02% H_3_PO_4_ in H_2_O and MeOH was used. Semi-preparative HPLC was performed on a Shimadzu LC-6AD using a C-18 column (YMC, 250 × 10 mm i.d., 5 μm; flow rate 4.0 mL·min^−1^).

The reagents used for UPLC-ESI-MS measurements were of HPLC grade and were obtained from Merck Inc. (Merck KGaA, Darmstadt, Germany). All other reagents were of analytical grade (Yuwang Reagent Company, Yucheng, Shandong, China). The purified water was prepared using a Millipore water purification system (Millipore, Bedford, MA, USA). Spectral grade solvents were used for spectroscopic measurements.

### 3.2. Collection of Oyster Shell Samples and Preparation for UPLC-MS Analysis

Samples were purchased from different drugstores, aquatic product markets and farms in China. Macroscopic identification was applied according to CP by Dr. Hong-bing Liu (School of Medicine and Pharmacy, Ocean University of China). Voucher specimens have been deposited in the School of Medicine and Pharmacy of Ocean University of China.

After milled to 40 mesh powder, each shell sample (100 g) was refluxed with MeOH for 1.5 h (500 mL, 3×) and the combined supernatant was further concentrated to dryness under reduced pressure. Prior to UPLC analysis, the MeOH extracts of oyster shells were dissolved in acetonitrile and filtered using a 0.22 μm filter.

### 3.3. UPLC-MS Conditions

Chemical profiles were recorded by UPLC-MS. The column temperature was maintained at 35 °C and the injection volume was fixed at 5 μL. The binary mobile phase consisted of solvent A, composed of 0.1% formic acid in water, and solvent B, which was 0.1% formic acid in acetonitrile. Separations were performed using a linear gradient of B into A at a flow rate of 0.4 mL·min^−1^ as follows: 0–0.5 min, 5–25% B; 0.5–3 min, 20–30% B; 3–5 min, 30–35% B; 5–10 min, 35–49% B; 10–14 min, 49–71% B; 14–20 min, 71–88% B; 20–23 min, 88–100% B, 23–26 min, 100% B. All the samples were kept at 10 °C during the analysis.

An ESI source with a V-spray interface was used, and each sample was analyzed in both positive and negative ionization modes. The ESI-MS conditions were optimized as follows: capillary voltage, 3 kV; cone voltage, 35 kV; source temperature, 100 °C; and desolvation temperature, 350 °C. Nitrogen was used as the desolvation gas and cone gas with the flow rate of 800 and 50 L·h^−1^, respectively. MS data were collected in the full scan mode from *m/z* 100 to 1000 amu over 0 to 26 min.

In order to ensure the stability and reliability of the analysis method, each sample was analyzed in triplicate.

### 3.4. Data Analysis

Chemical profiles were represented by a BPI chromatogram. All the raw data were processed using the MassLynx V4.1 software with a MarkerLynx program (Waters Corp.: Milford, MA, USA, 2009), which allows the detection of the mass, retention time and intensity of the peak eluted in each chromatogram. The resulting three-dimensional data, containing retention time-extract mass pairs and normalized ion intensities were automatically exported to SIMCA-P 11.5 software (Umetrics: Umea, Sweden, 2009) for further multivariate statistical analysis by PCA and OPLS-DA.

### 3.5. Separation of Caffeine (1)

The large-scale of crude drug of *Ostreae concha* was purchased from An-guo drugmarket, Hebei province, which was identified as *C. gigas* according to morphological attributes. For initial analysis of the natural products, 37.5 kg milled crude drug was extracted with 80% EtOH and then desalted by CH_2_Cl_2_ extraction. The CH_2_Cl_2_ extract (1.64 g) was fractionated by Sephadex LH-20 (GE Healthcare, Sweden) eluting with CH_2_Cl_2_-MeOH (1:1) to yield 4 fractions. Fraction 2 (0.87 g) was repeatedly separated by Sephadex LH-20 eluting with MeOH and further by semi-preparative HPLC (MeOH·H_2_O, 25:75) to give compound **1** (7.4 mg, 1 μM per kg).

## 4. Conclusions

The results of this study allow a clear discrimination of the chemical characteristics among perennial medicinal shells and cultured shells based using UPLC-MS data and chemometric analysis. Considering the differences between the cultured and medicinal shells, it is considered inappropriate to replace medicinal shells with the cultured shells as an alternative resource. Further chemical works on minor inorganic elements and bioassay tests are needed to evaluate the potential medicinal values of the cultured shells. Caffeine was isolated and identified as a key compound in medicinal materials of oyster shell,but its biological origin should be investigated. Clarification of more key compounds needs further study using appropriate multi-technologies. This study preliminarily provided the basis for a needed quality standard of marine-derived TCM *Ostreae concha*.

## Supplementary Files

Supplementary File 1: PDF-Document (PDF, 1147 KB)
