# Supplementary material for: Chemical Profiles and Identification of Key Compound Caffeine in Marine-Derived Traditional Chinese Medicine Ostreae concha
Source: Mar Drugs. 2012 May 23;10(5):1180–91. doi: 10.3390/md10051180 (PMC3397450; doi:10.3390/md10051180)

Supplementary Materials

Figure S1. The positive BPI chromatogram of the sample A1 ……...S3
Figure S2. The negative BPI chromatogram of the sample A1 ……..S3
Figure S3. The positive BPI chromatogram of the sample A2 ……...S4
Figure S4. The negative BPI chromatogram of the sample A2……...S4
Figure S5. The positive BPI chromatogram of the sample A3 ……...S5
Figure S6. The negative BPI chromatogram of the sampleA3 ……...S5
Figure S7 The positive BPI chromatogram of the sample A4……..S6
Figure S8. The negative BPI chromatogram of the sample A4 ……..S6
Figure S9. The positive BPI chromatogram of the sample A5 ……...S7
Figure S10. The negative BPI chromatogram of the sample A5 ……..S7
Figure S11. The positive BPI chromatogram of the sample A6 ……...S8
Figure S12. The negative BPI chromatogram of the sample A6 ……..S8
Figure S13. The positive BPI chromatogram of the sample A7 ……...S9
Figure S14. The negative BPI chromatogram of the sample A7 ……..S9
Figure S15. The positive BPI chromatogram of the sample A8 ……...S10
Figure S16. The negative BPI chromatogram of the sample A8 ……..S10
Figure S17. The positive BPI chromatogram of the sample A9 ……...S11
Figure S18. The negative BPI chromatogram of the sample A9 ……..S11
Figure S19. The positive BPI chromatogram of the sample A10……...S12
Figure S20. The negative BPI chromatogram of the sample A10 ……..S12
Figure S21. The negative BPI chromatogram of the sample A11 ……………………..…………S13
Figure S22. The positive BPI chromatogram of the sample A12 ……..S13
Figure S23. The negative BPI chromatogram of the sample A12 ……..S14
Figure S24. The positive BPI chromatogram of the sample B1 ……...S14
Figure S25. The negative BPI chromatogram of the sample B1 ……..S15
Figure S26. The positive BPI chromatogram of the sample B2 ……...S15
Figure S27. The negative BPI chromatogram of the sample B2 ……..S16
Figure S28. The positive BPI chromatogram of the sample B3 ……...S16
Figure S29. The negative BPI chromatogram of the sample B3 ……..S17
Figure S30. The positive BPI chromatogram of the sample B4 ……...S17
Figure S31. The negative BPI chromatogram of the sample B4 ……..S18
Figure S32. The positive BPI chromatogram of the sample B5 ……...S18
Figure S33. The negative BPI chromatogram of the sample B5 ....…..S19
Figure S34. The positive BPI chromatogram of the sample B6 ……...S19
Figure S35. The negative BPI chromatogram of the sample B6 ……………………...……S20
Figure S36. The positive BPI chromatogram of the sample B7 ……...S20
Figure S37. The negative BPI chromatogram of the sample B7 ………………………………....S21
Figure S38. The positive BPI chromatogram of the sample B8 ……...S21
Figure S39. The negative BPI chromatogram of the sample B8 ……..S22
Figure S40. The positive BPI chromatogram of the sample B9 ……...S22
Figure S41. The negative BPI chromatogram of the sample B9………………………………….S23

Figure S42. The positive BPI chromatogram of the sample B10 ……….......................................S23
Figure S43. The negative BPI chromatogram of the sample B10 ……………………..…….……S24
Figure S44. The positive BPI chromatogram of the sample B11……….......................................S24
Figure S45. The negative BPI chromatogram of the sample B11 …...S25
Figure S46. The positive BPI chromatogram of the sample B12 ……...S25
Figure S47. The negative BPI chromatogram of the sample B12 …...S26
Figure S48. The OPLS-DA score plot in positive ion mode…………………………….………...…S26
Figure S49. The ^{1}H-NMR spectrum of compound **1** in DMSO-d_6……………………………...….S27
Figure S50. The ^{13}C-NMR spectrum of compound **1** in DMSO-d_6………………………………S28
Figure S51. The HMQC spectrum of compound **1** in DMSO-d_6…………………………………..S29
Figure S52. The HMBC spectrum of compound **1** in DMSO-d_6……………………….……………S30

Figure S1. The positive BPI chromatogram of the sample A1

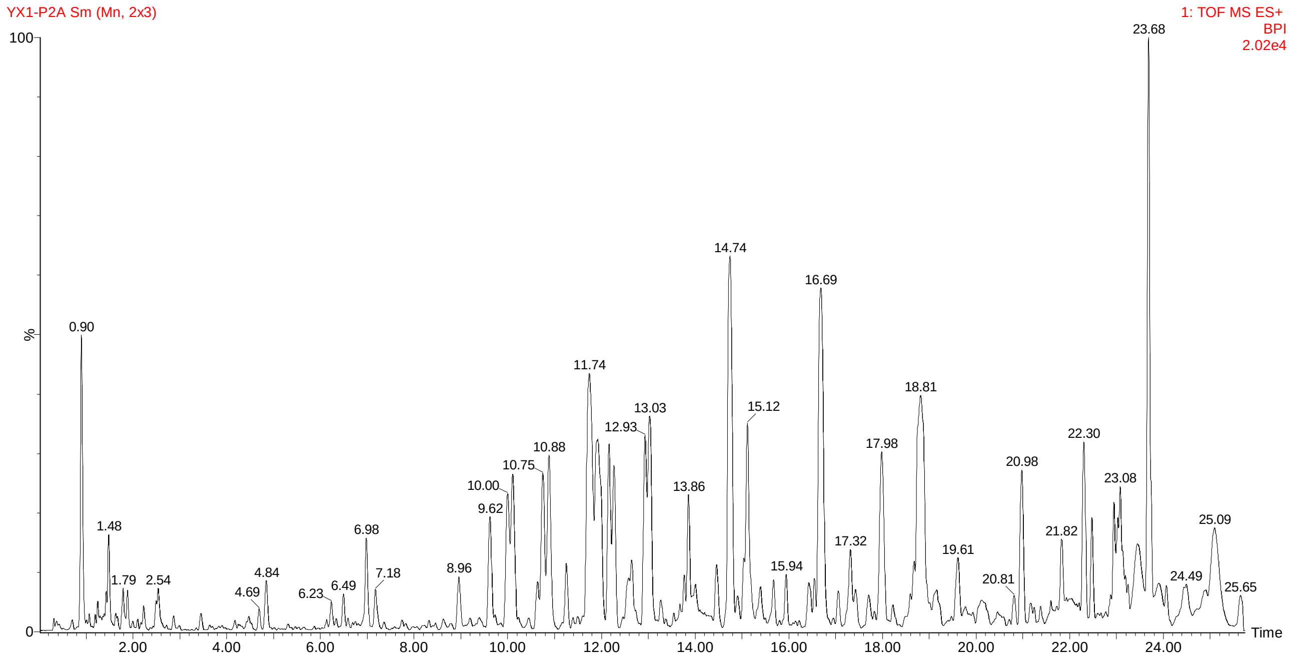

Figure S2. The negative BPI chromatogram of the sample A1

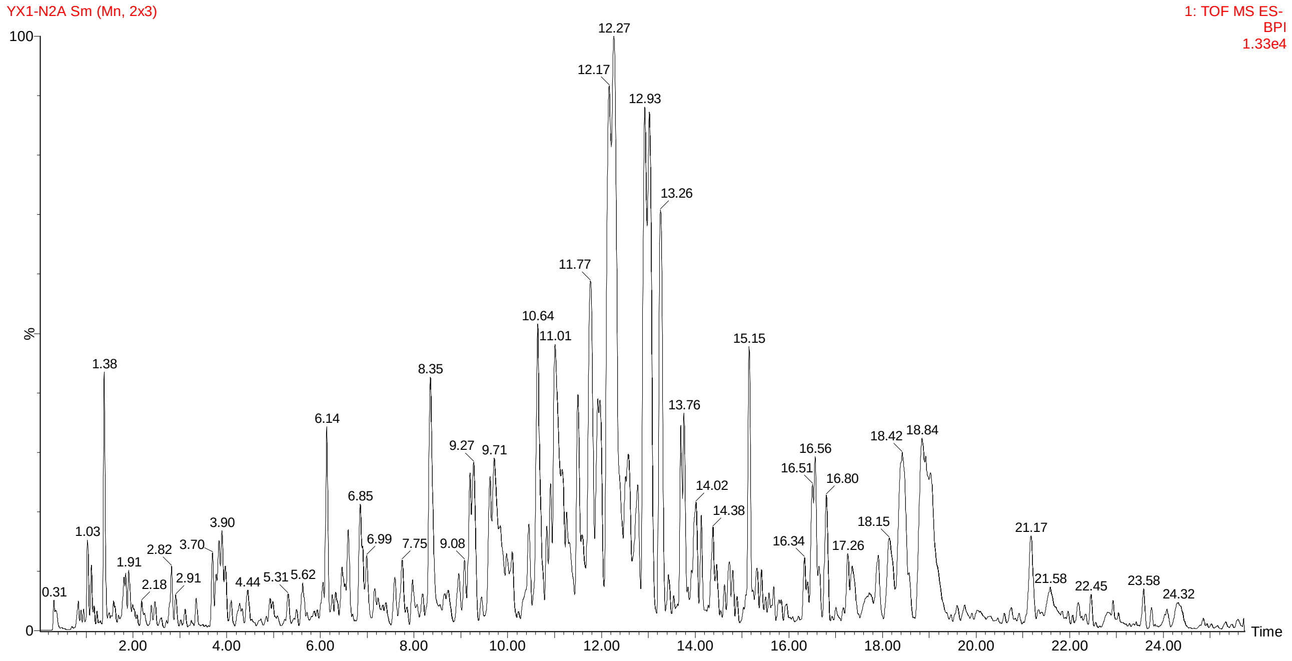

Figure S3. The positive BPI chromatogram of the sample A2

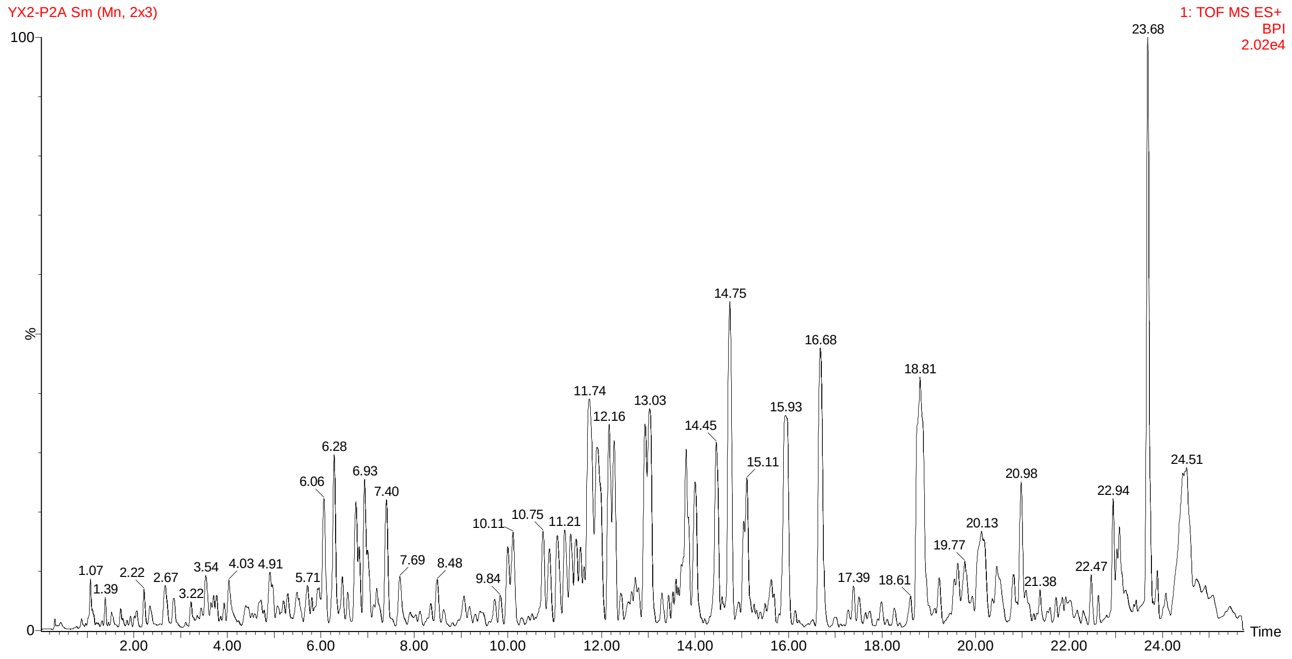

Figure S4. The negative BPI chromatogram of the sample A2

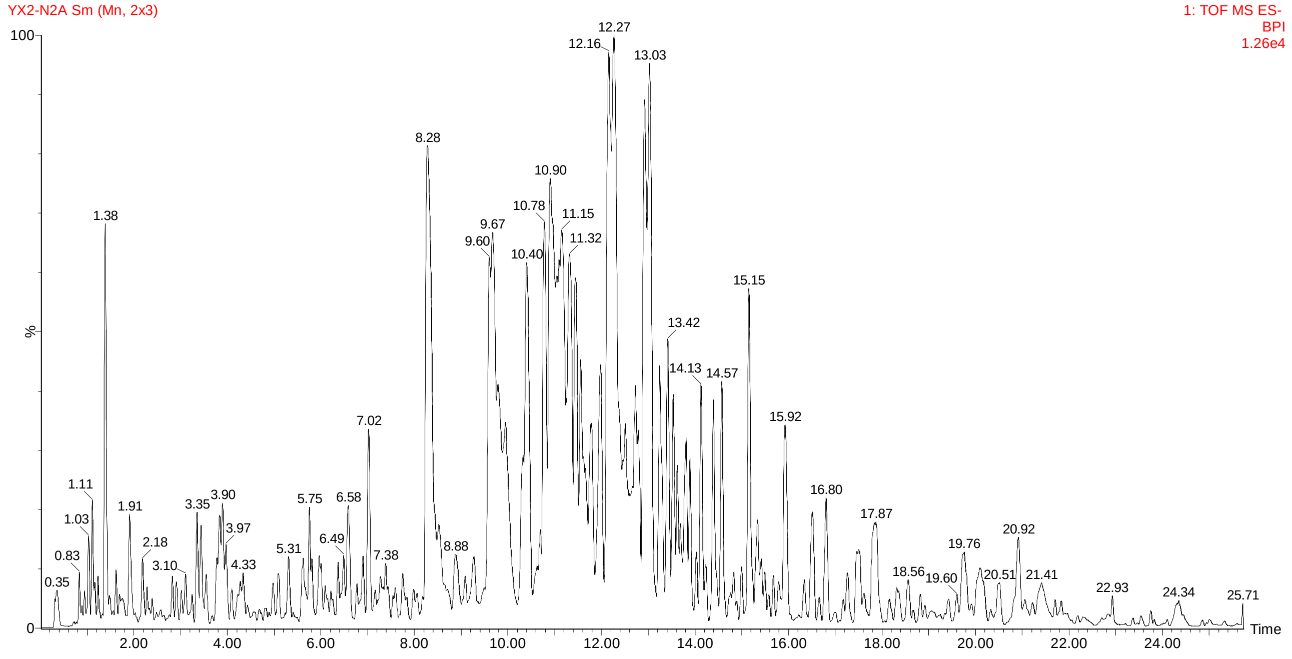

Figure S5. The positive BPI chromatogram of the sample A3

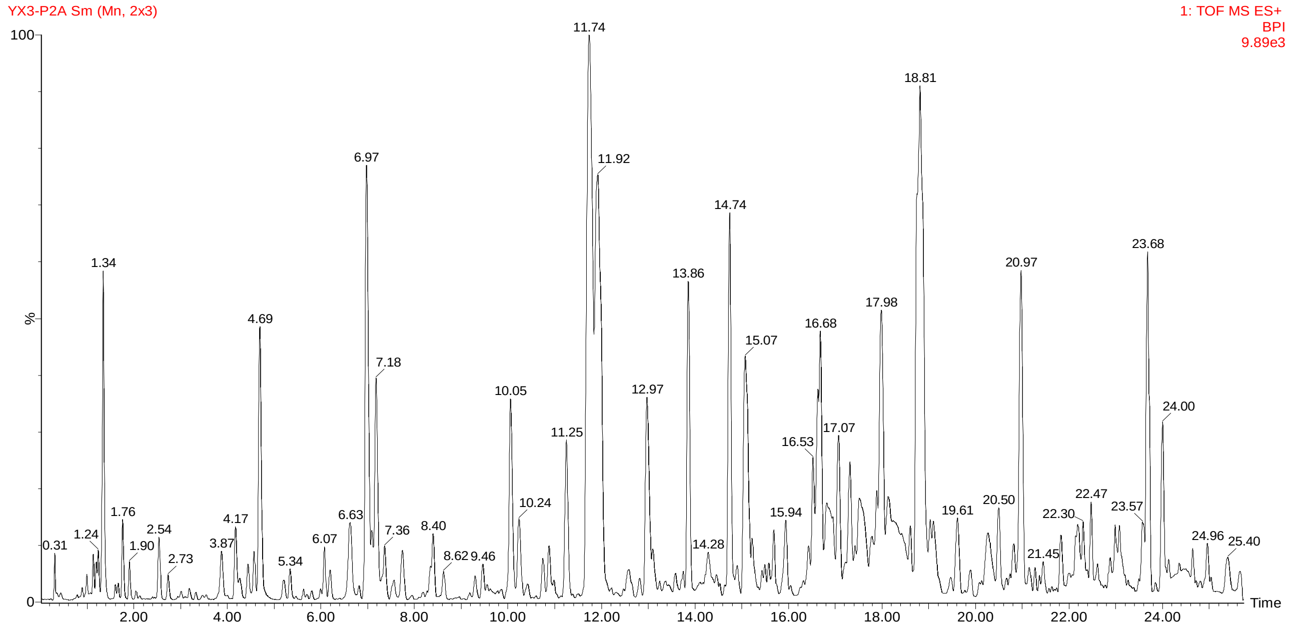

Figure S6. The negative BPI chromatogram of the sampleA3

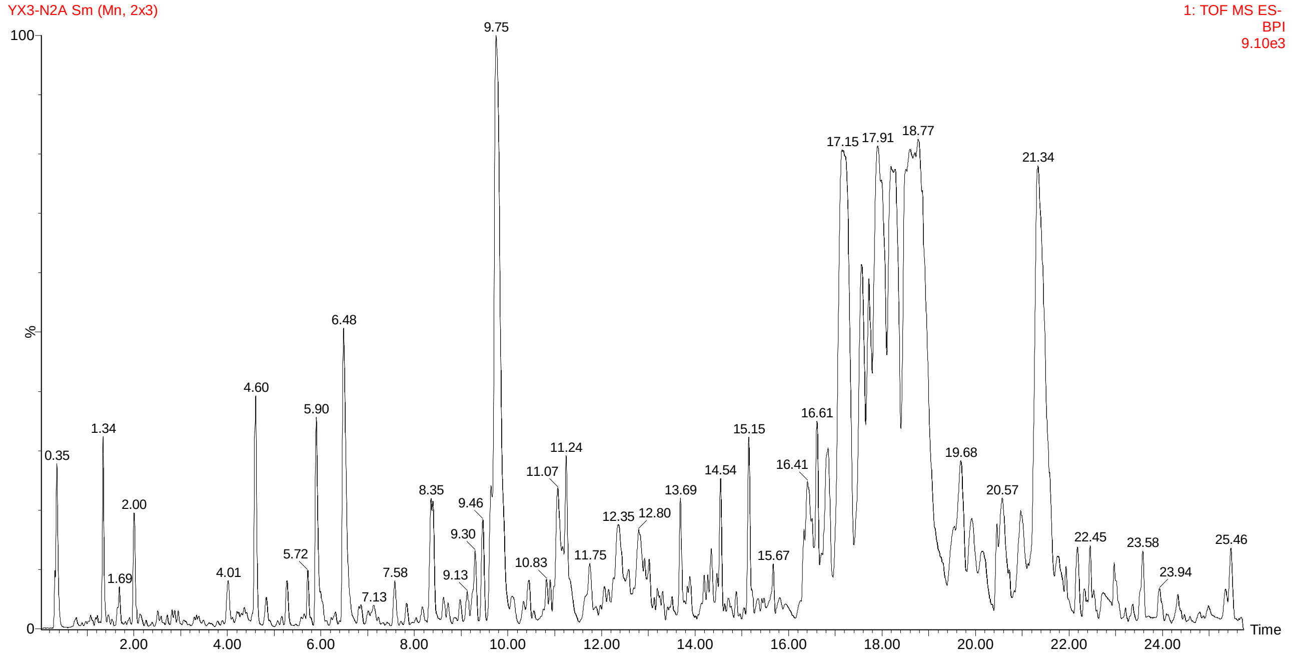

Figure S7 The positive BPI chromatogram of the sample A4

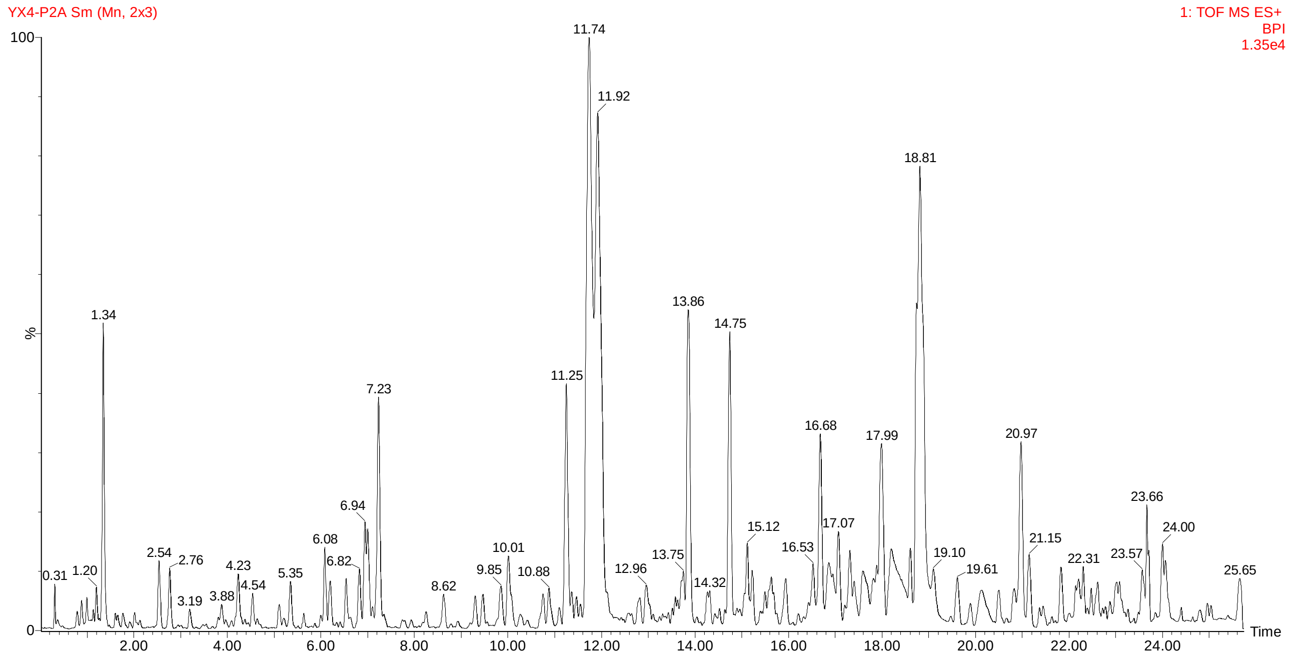

Figure S8. The negative BPI chromatogram of the sample A4

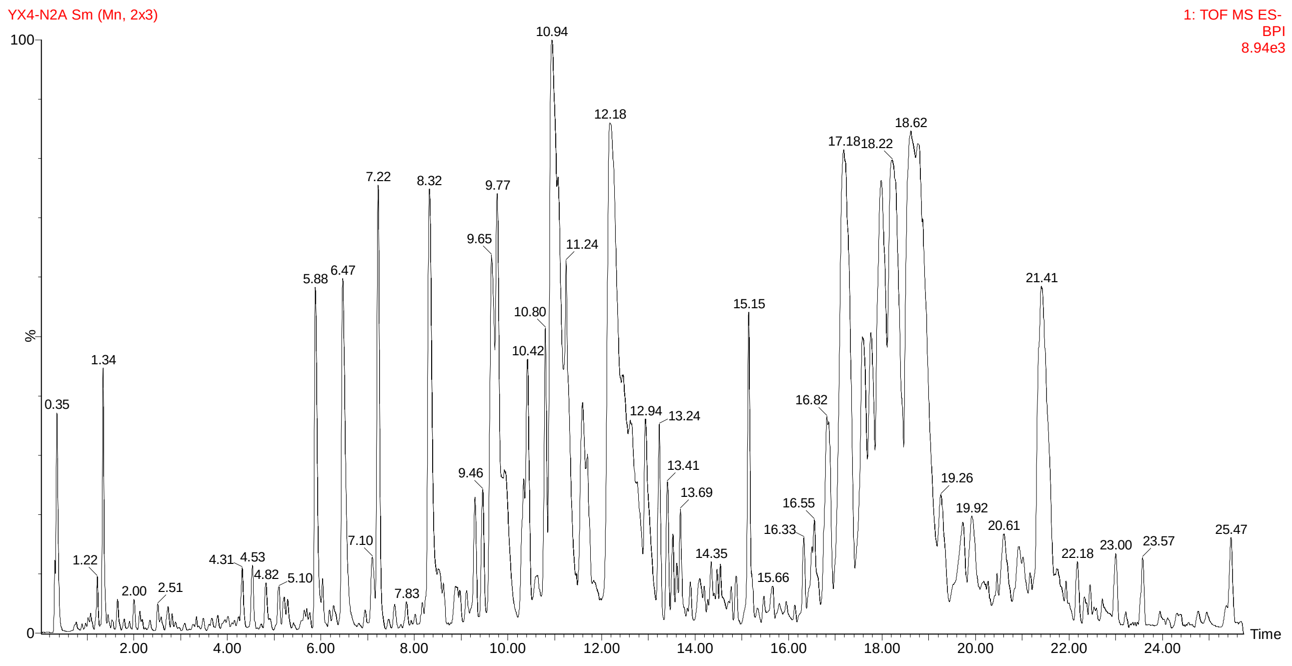

Figure S9. The positive BPI chromatogram of the sample A5

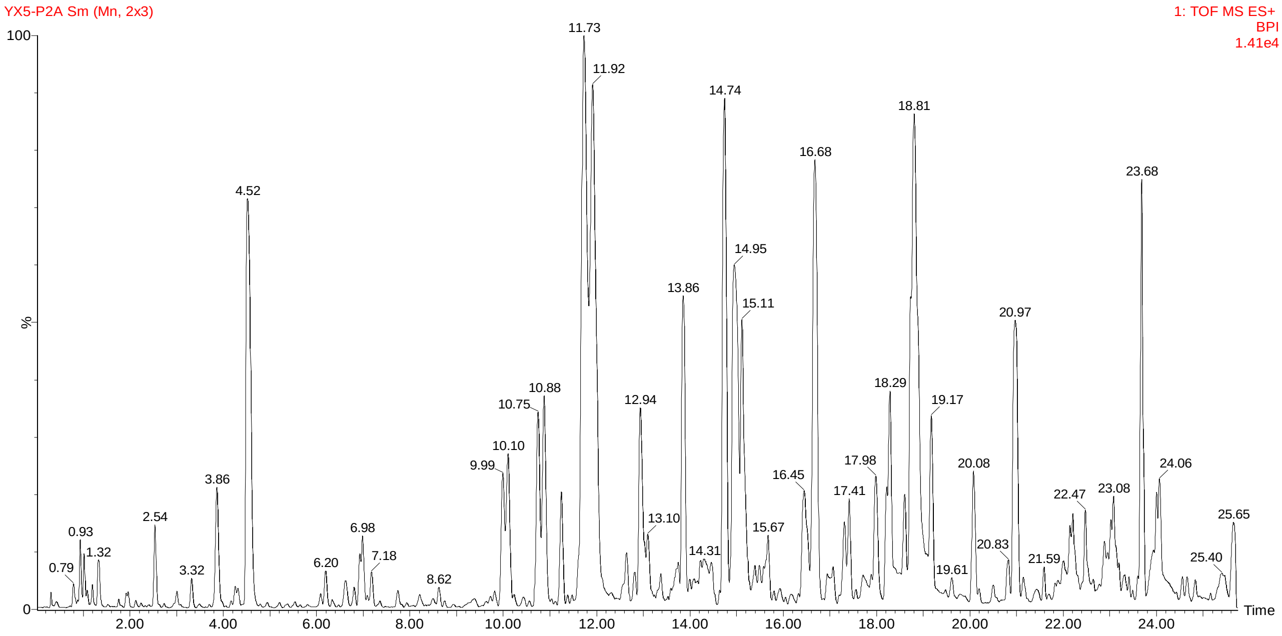

Figure S10. The negative BPI chromatogram of the sample A5

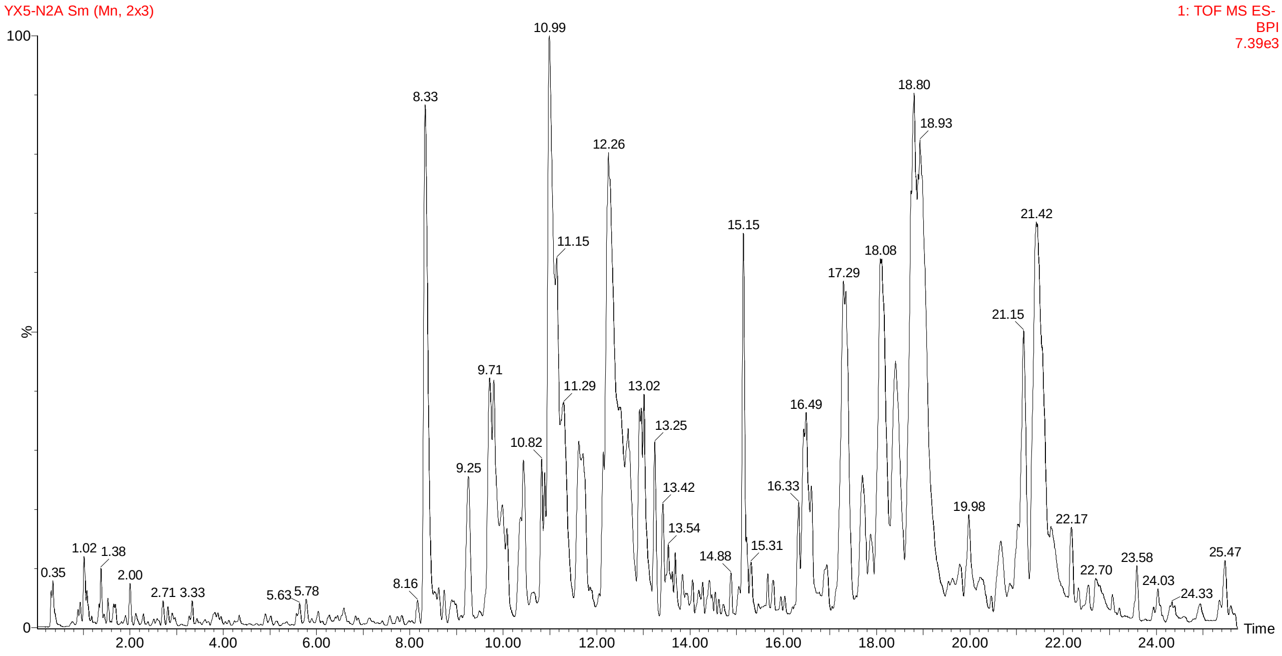

Figure S11. The positive BPI chromatogram of the sample A6

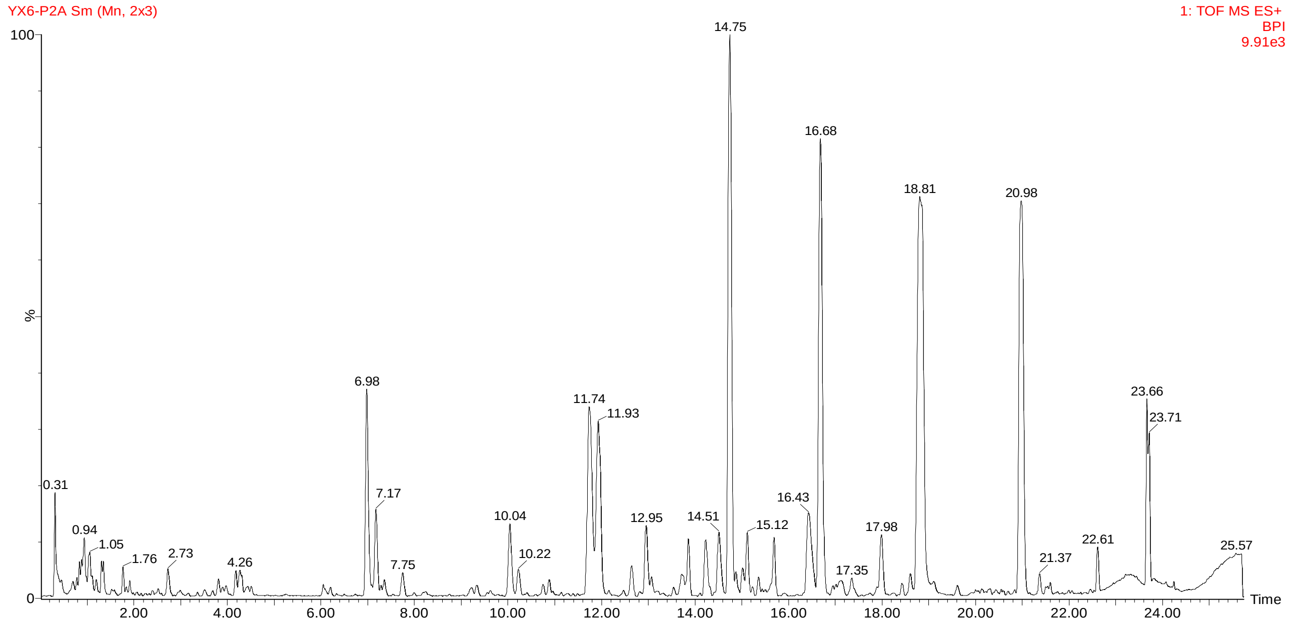

Figure S12. The negative BPI chromatogram of the sample A6

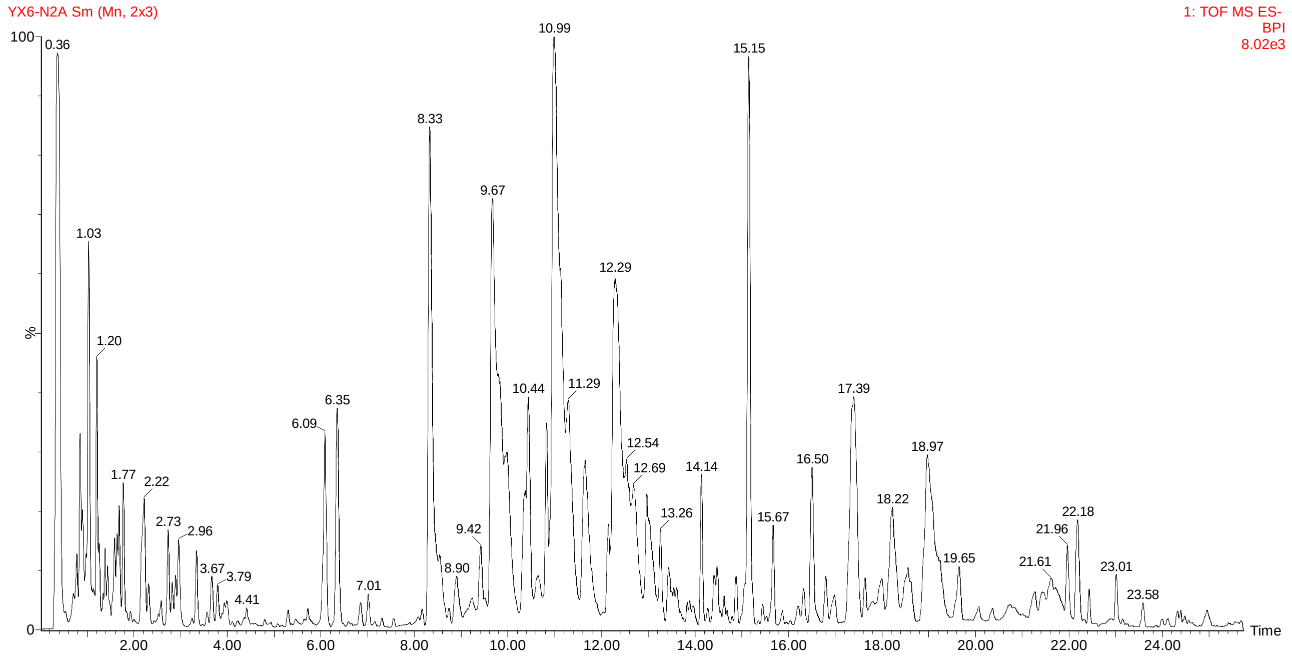

Figure S13. The positive BPI chromatogram of the sample A7

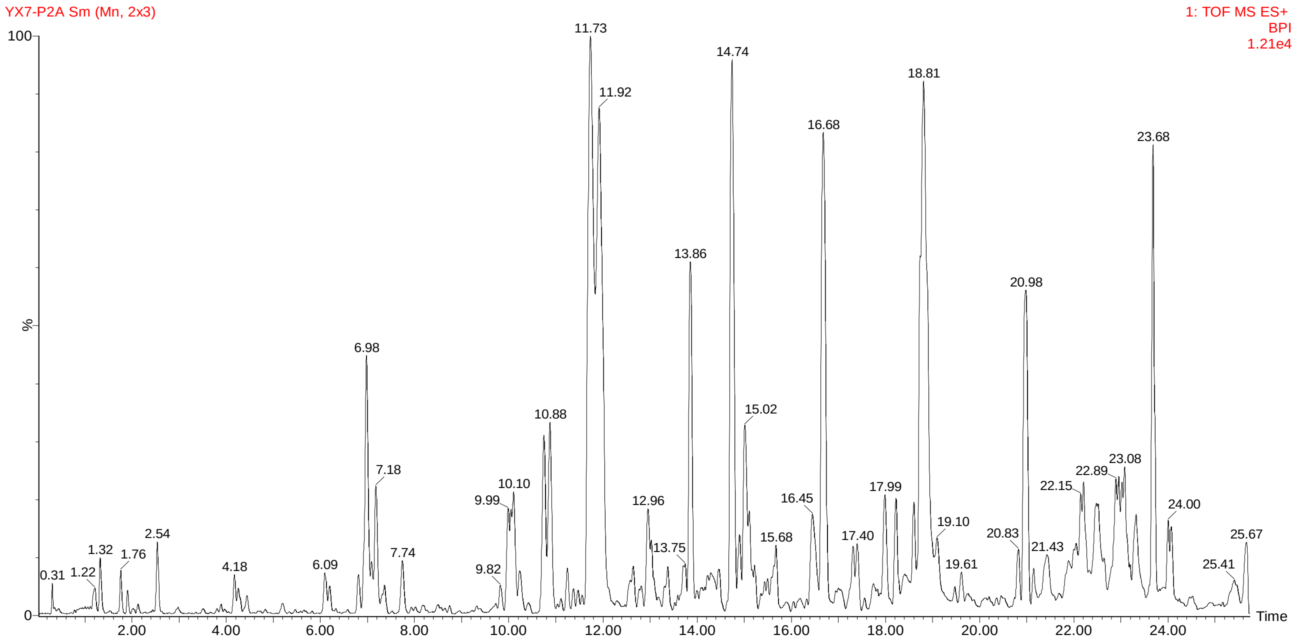

Figure S14. The negative BPI chromatogram of the sample A7

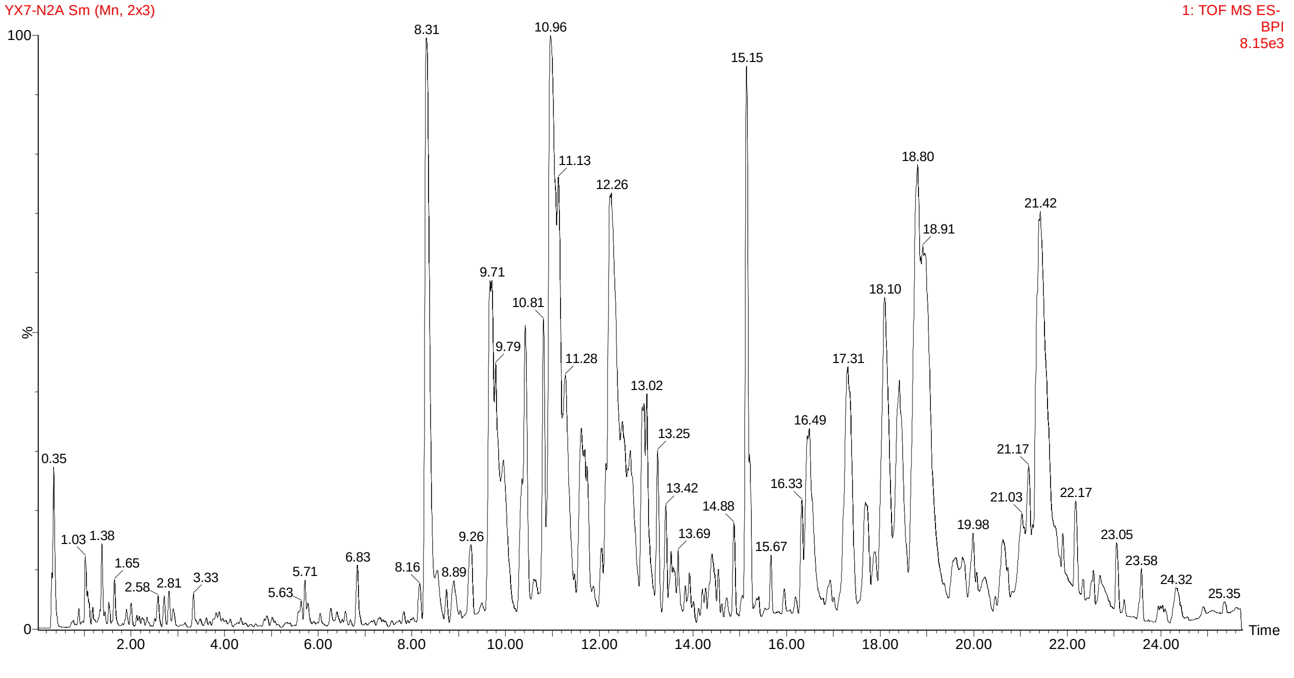

Figure S15. The positive BPI chromatogram of the sample A8

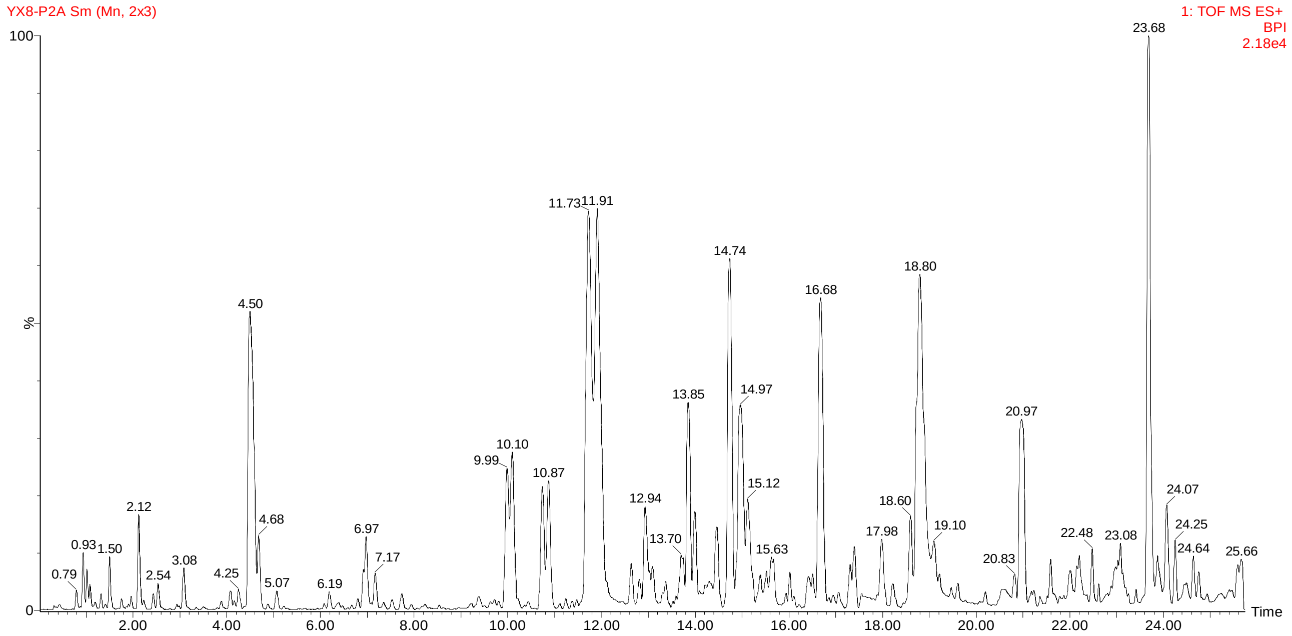

Figure S16. The negative BPI chromatogram of the sample A8

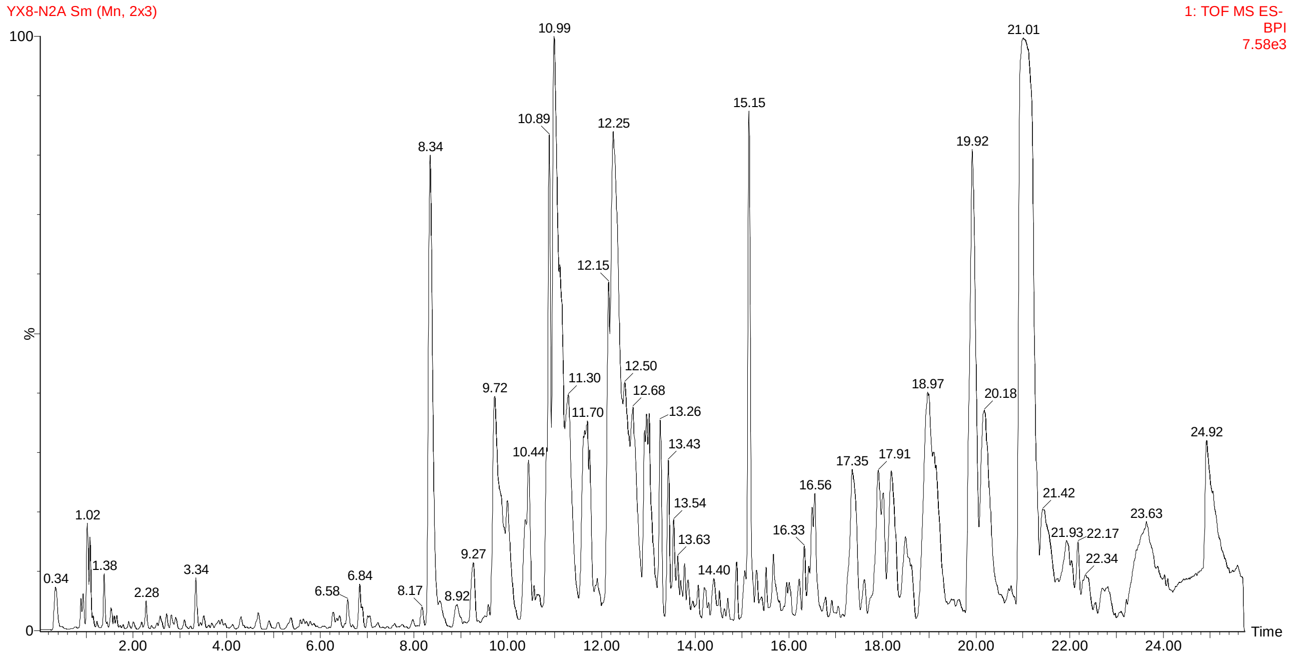

Figure S17. The positive BPI chromatogram of the sample A9

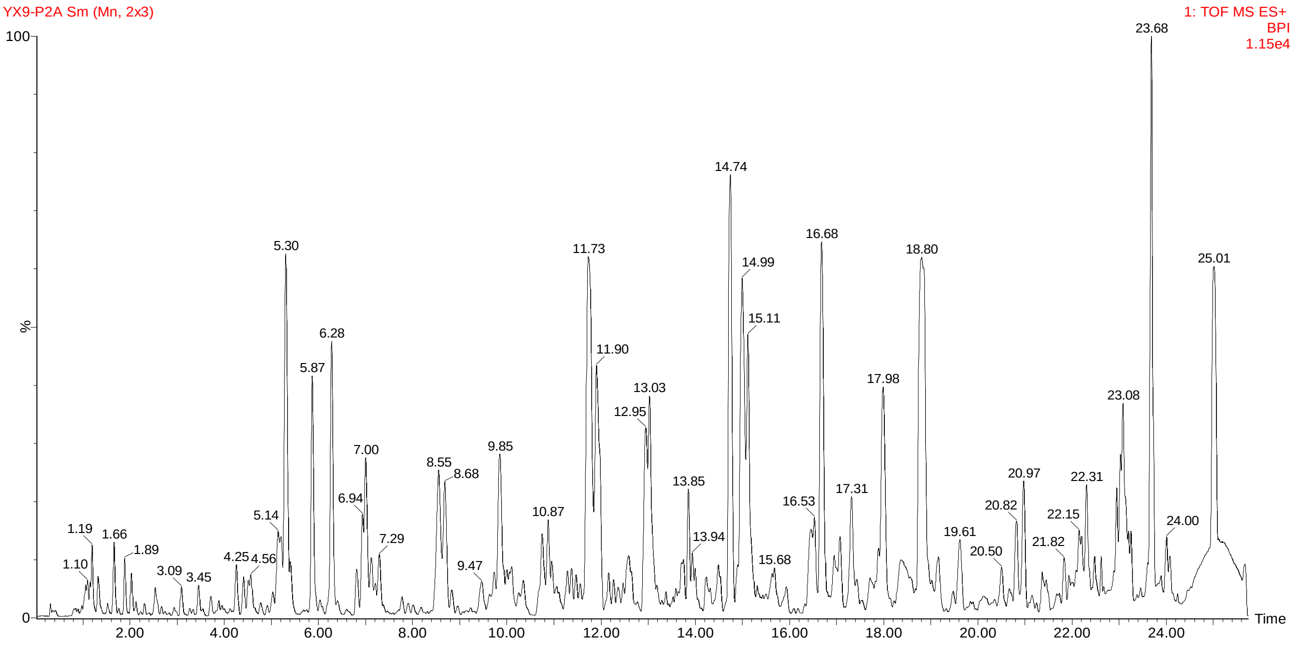

Figure S18. The negative BPI chromatogram of the sample A9

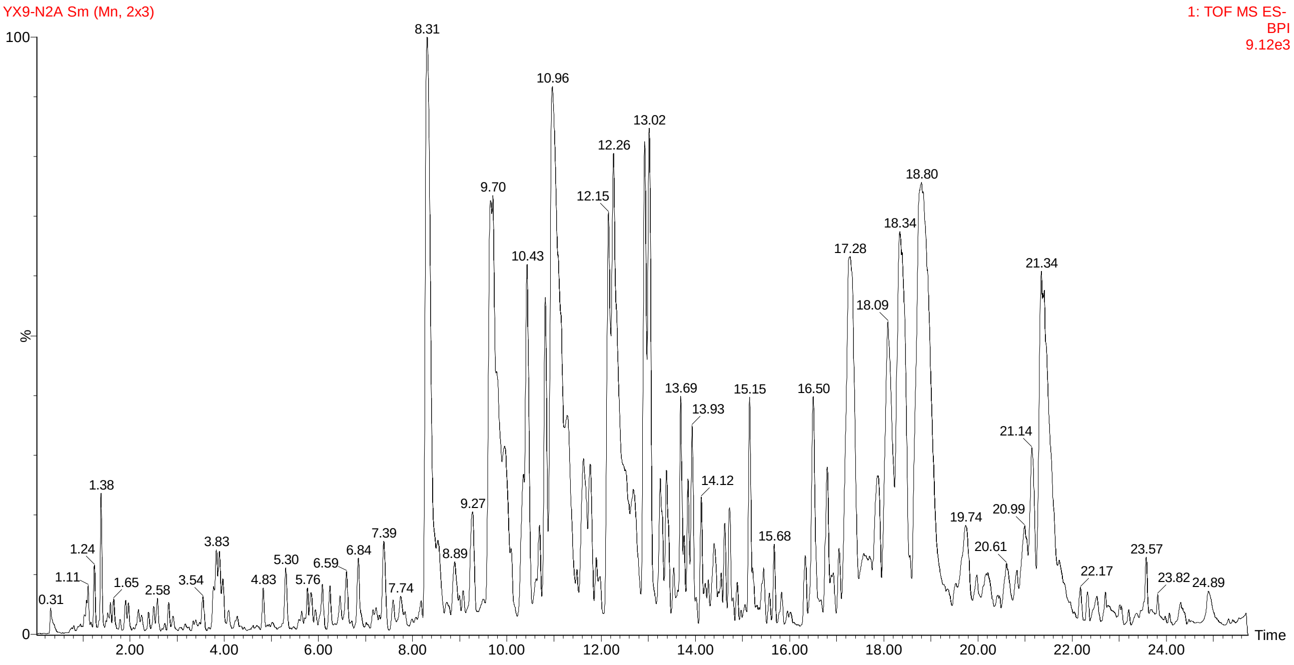

Figure S19. The positive BPI chromatogram of the sample A10

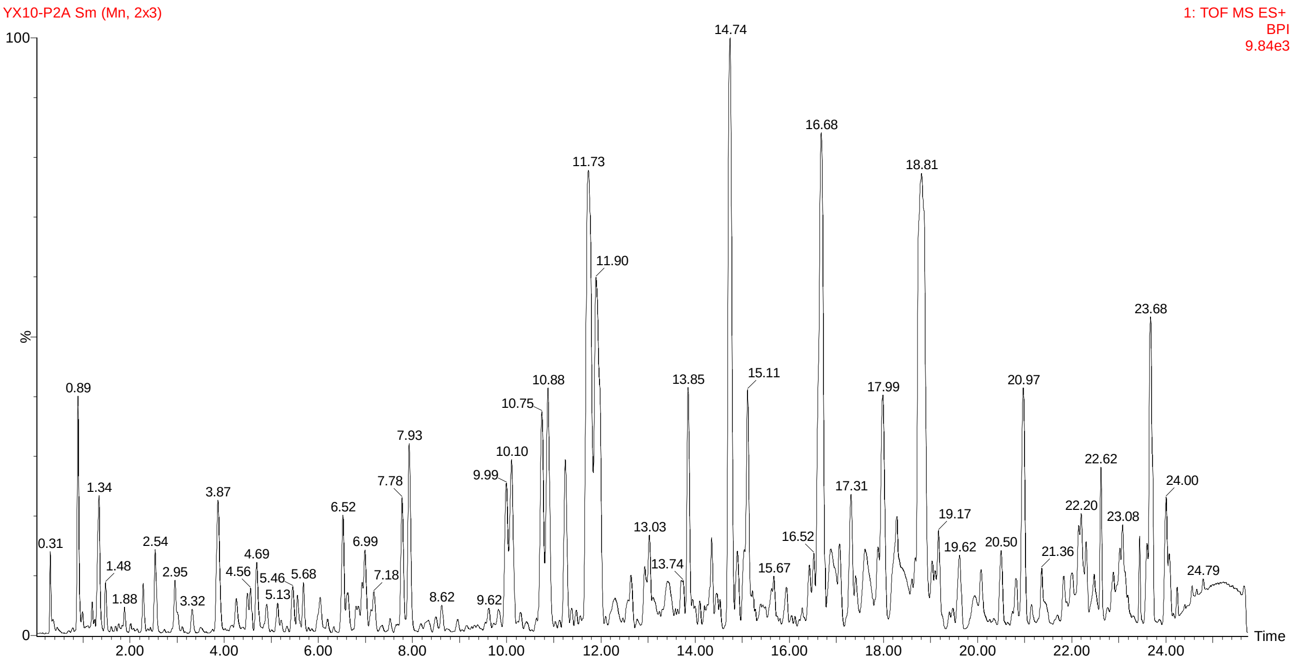

Figure S20. The negative BPI chromatogram of the sample A10

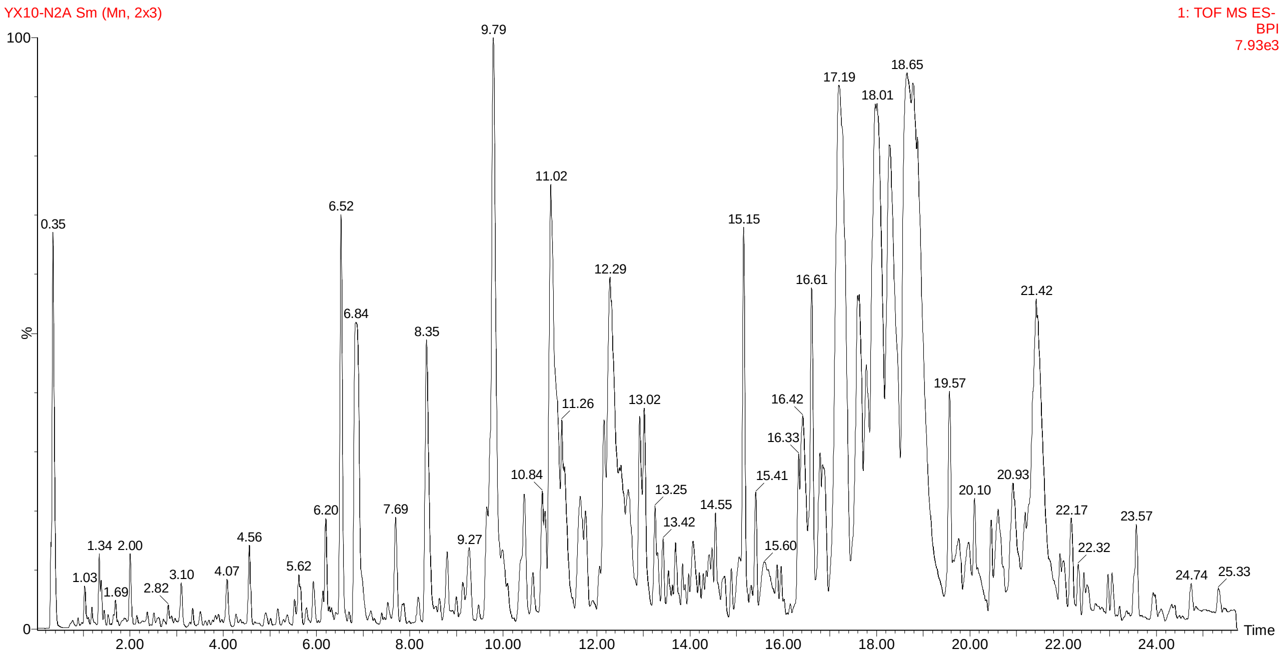

Figure S21. The negative BPI chromatogram of the sample A11

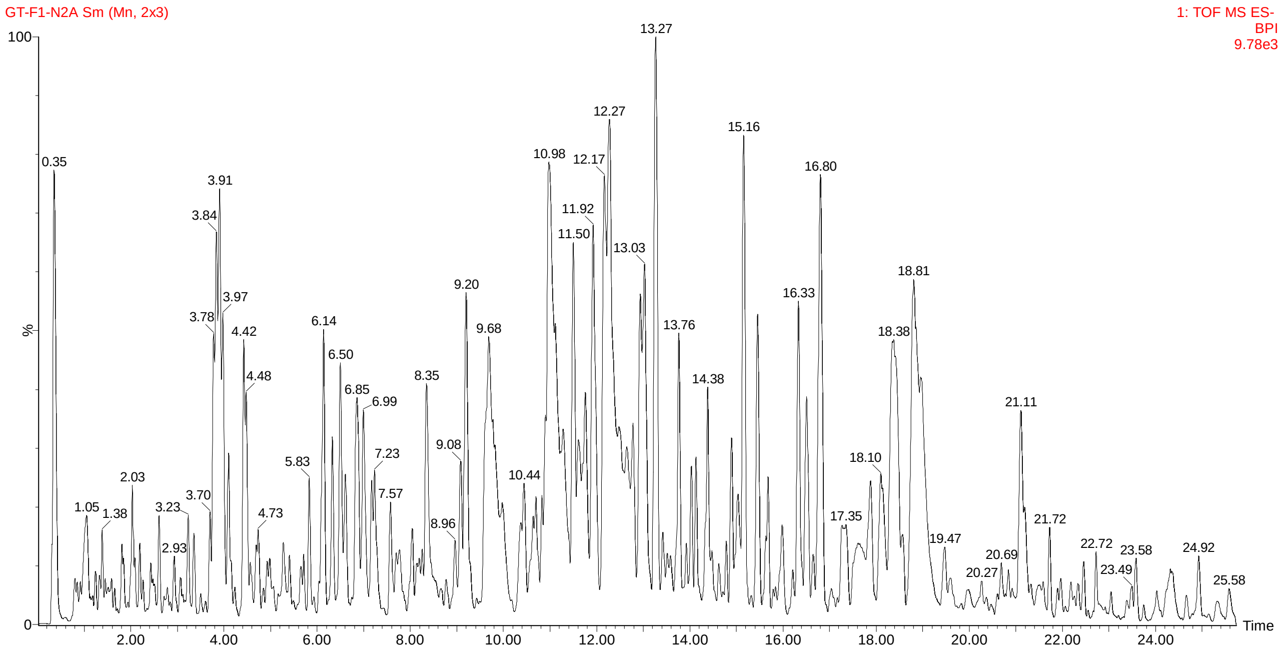

Figure S22. The positive BPI chromatogram of the sample A12

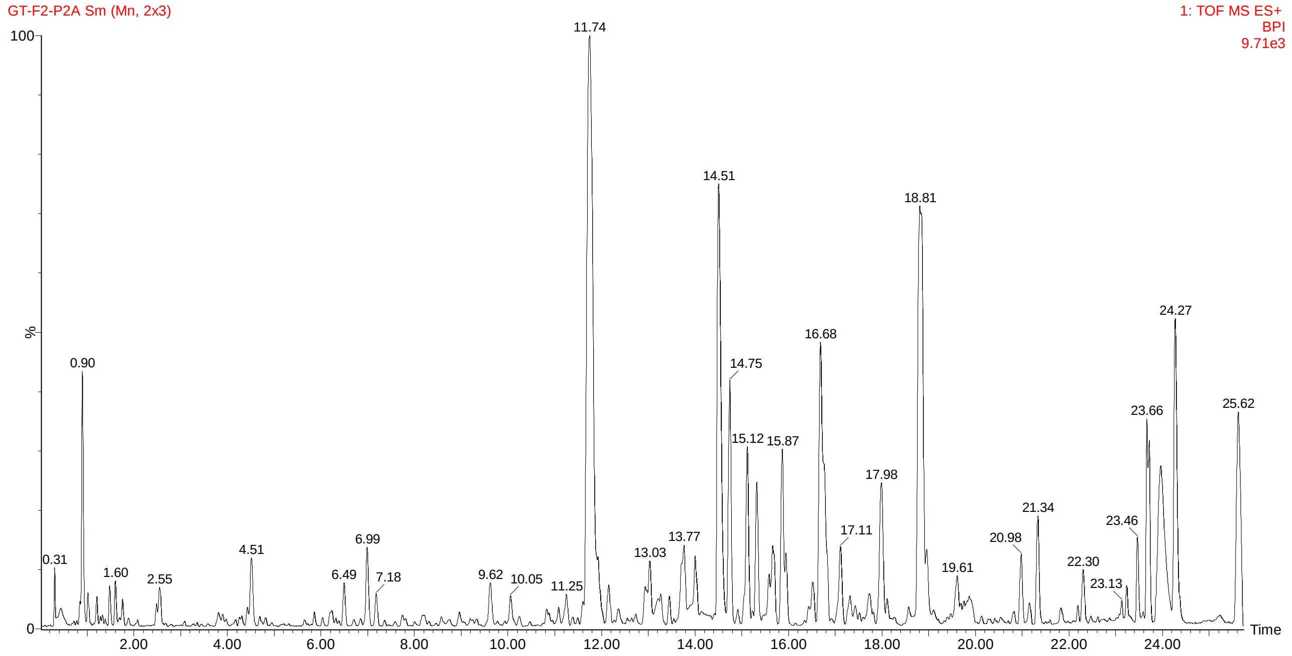

Figure S23. The negative BPI chromatogram of the sample A12

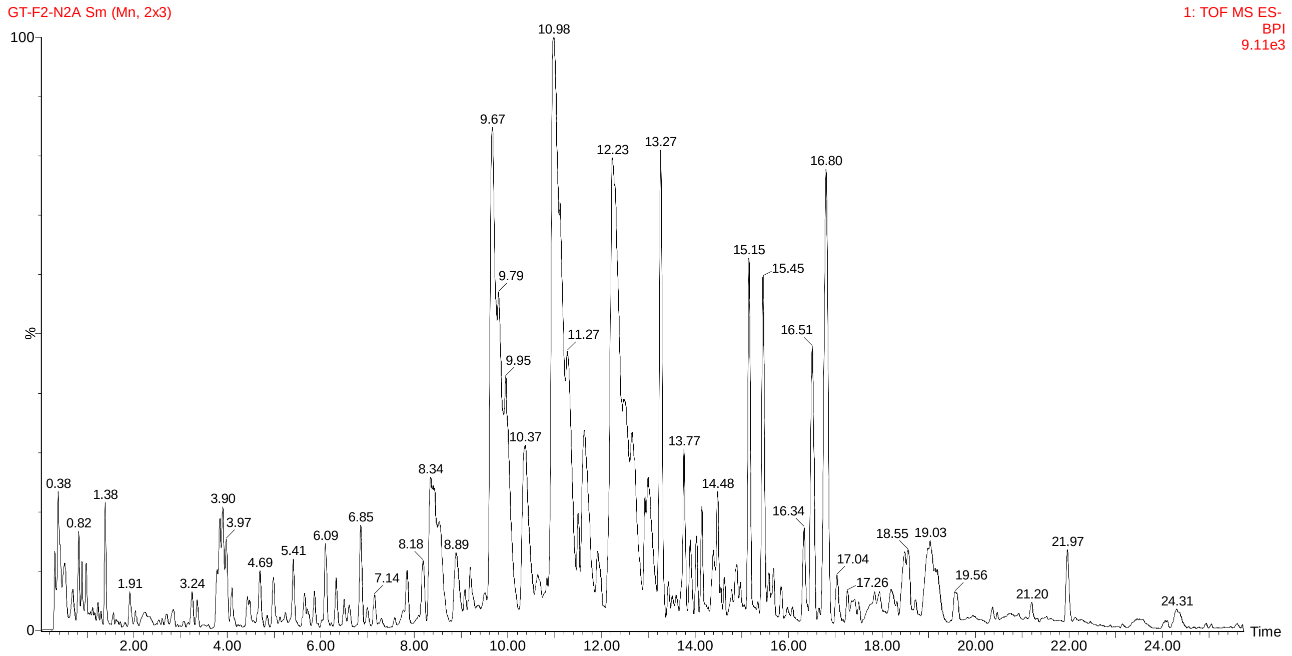

Figure S24. The positive BPI chromatogram of the sample B1

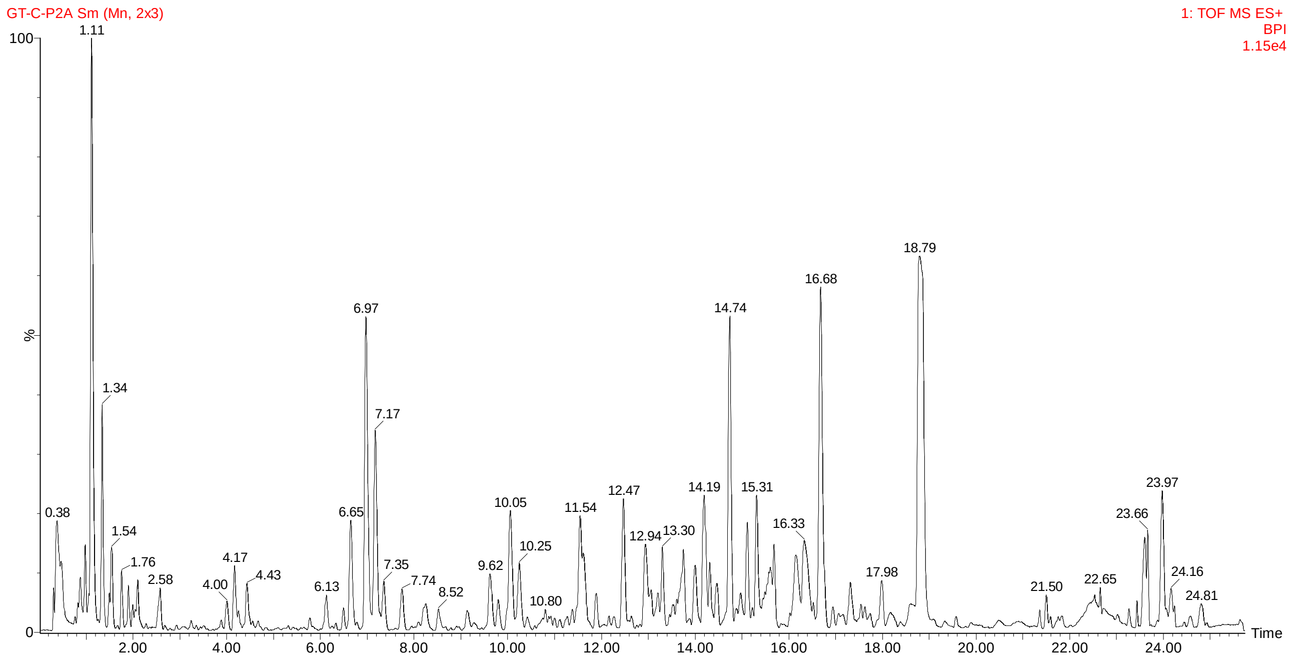

Figure S25. The negative BPI chromatogram of the sample B1

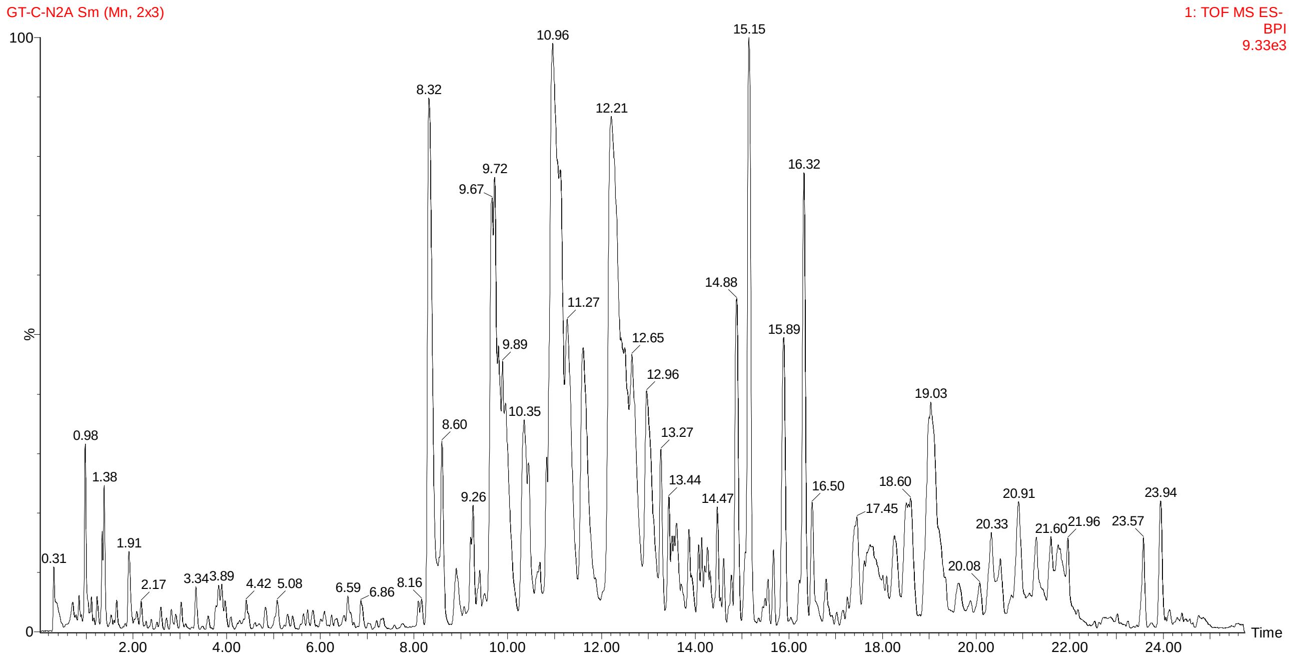

Figure S26. The positive BPI chromatogram of the sample B2

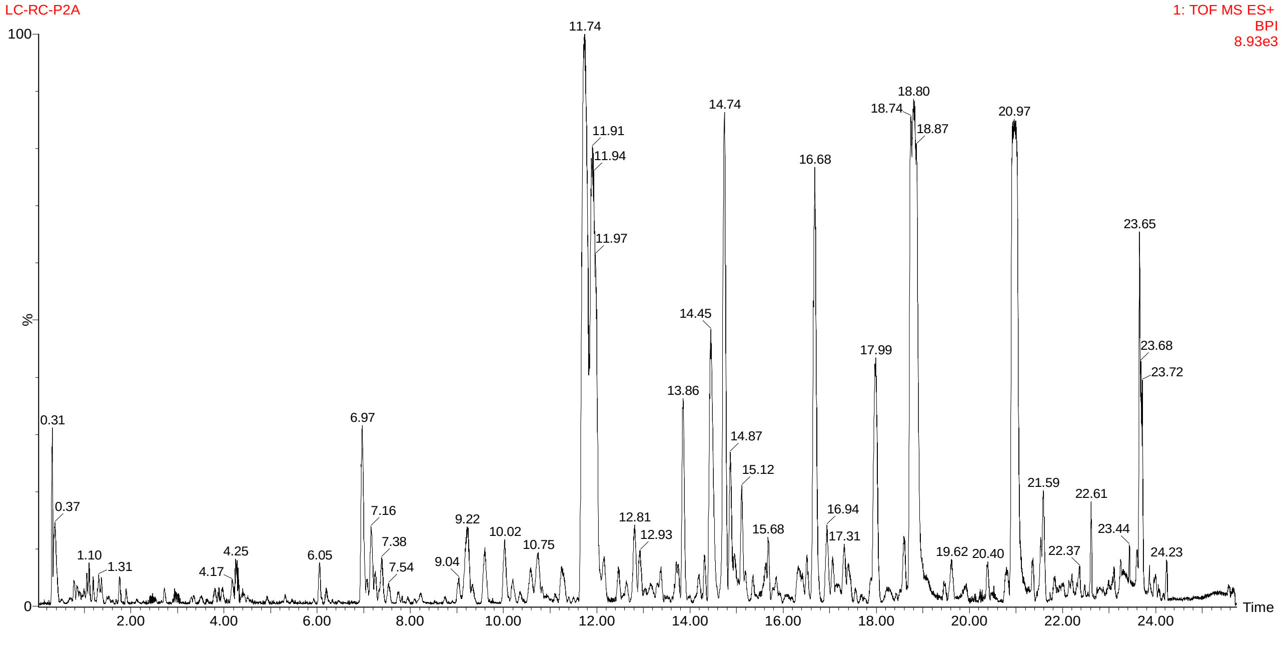

Figure S27. The negative BPI chromatogram of the sample B2

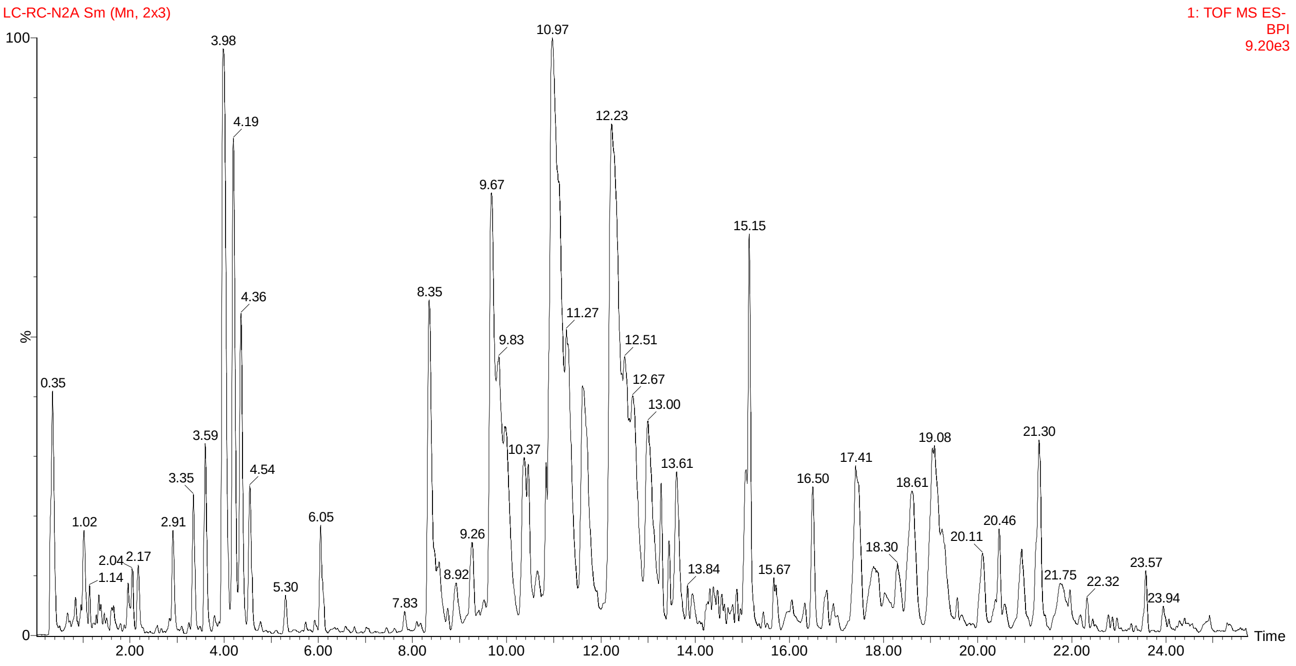

Figure S28. The positive BPI chromatogram of the sample B3

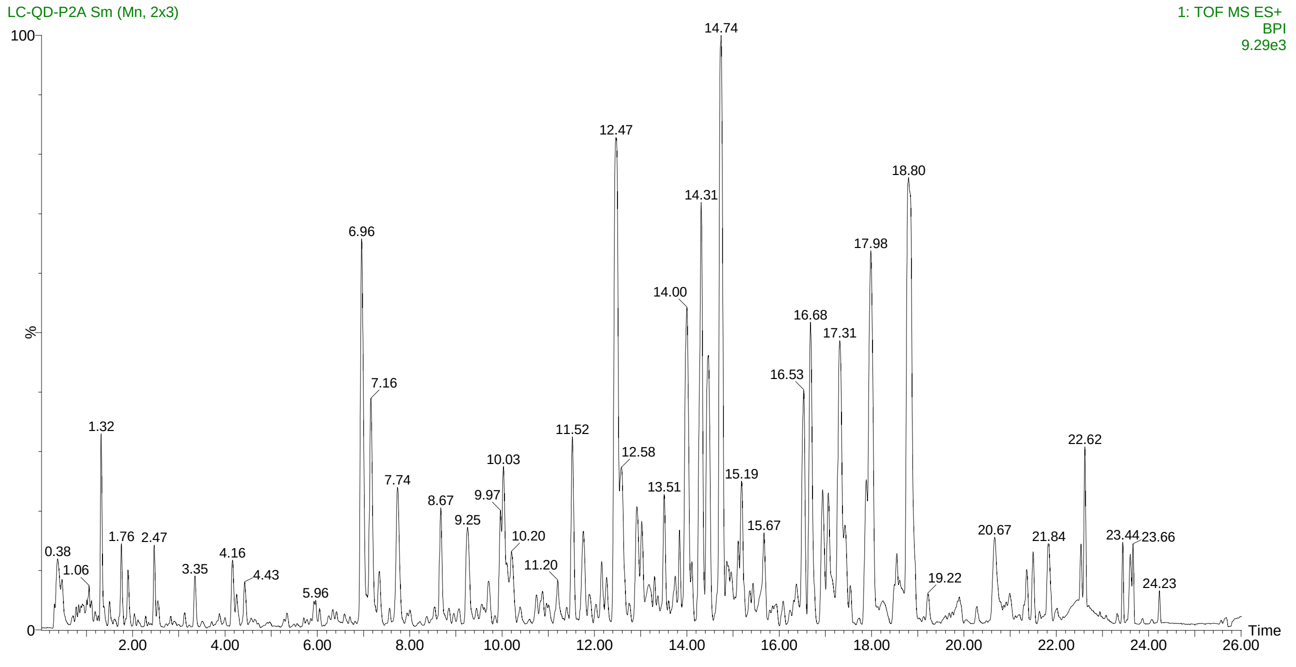

Figure S29. The negative BPI chromatogram of the sample B3

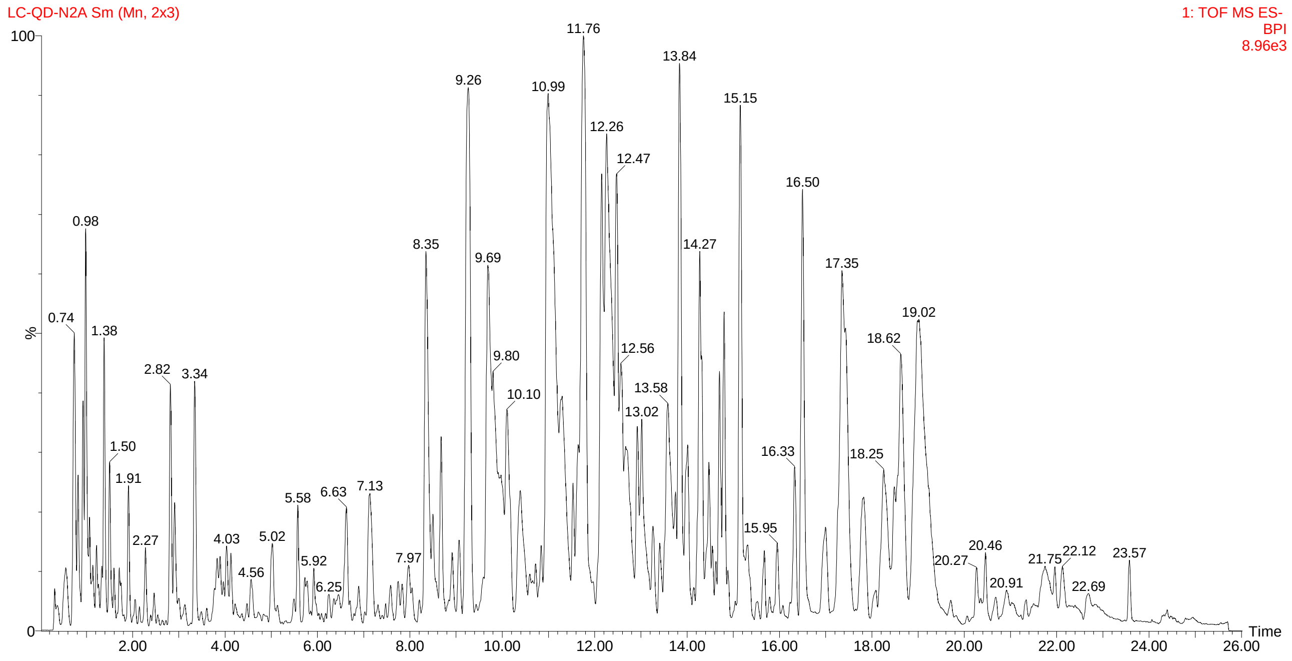

Figure S30. The positive BPI chromatogram of the sample B4

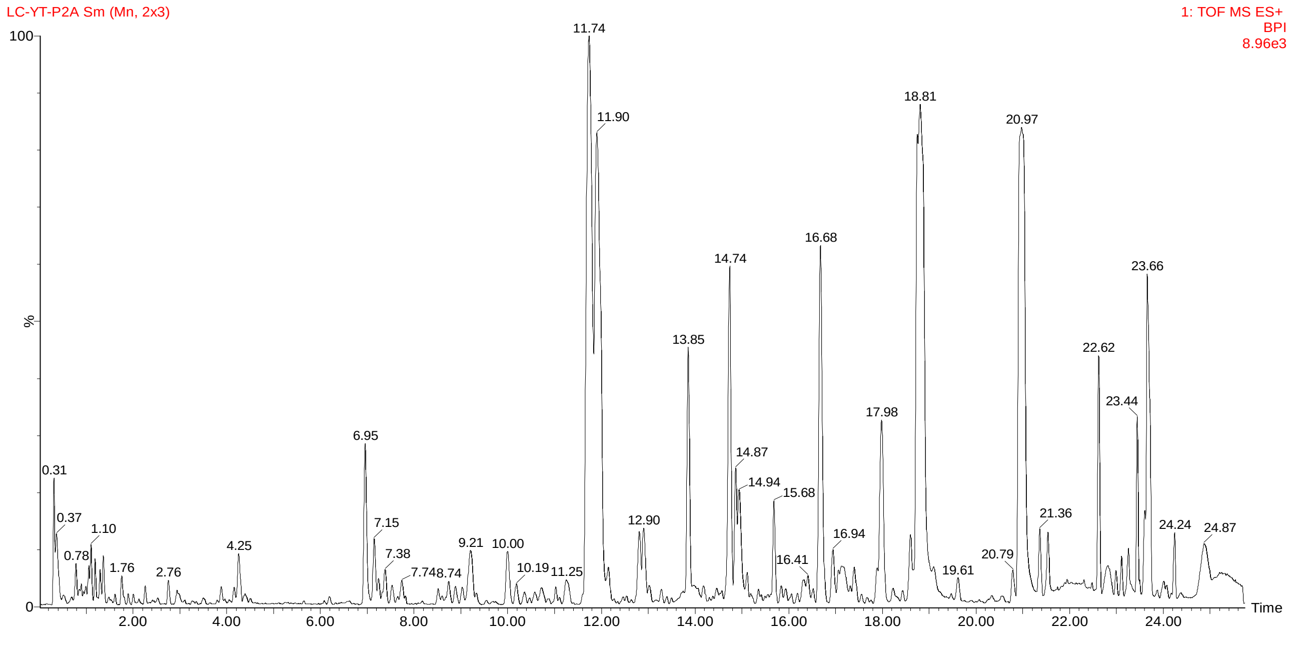

Figure S31. The negative BPI chromatogram of the sample B4

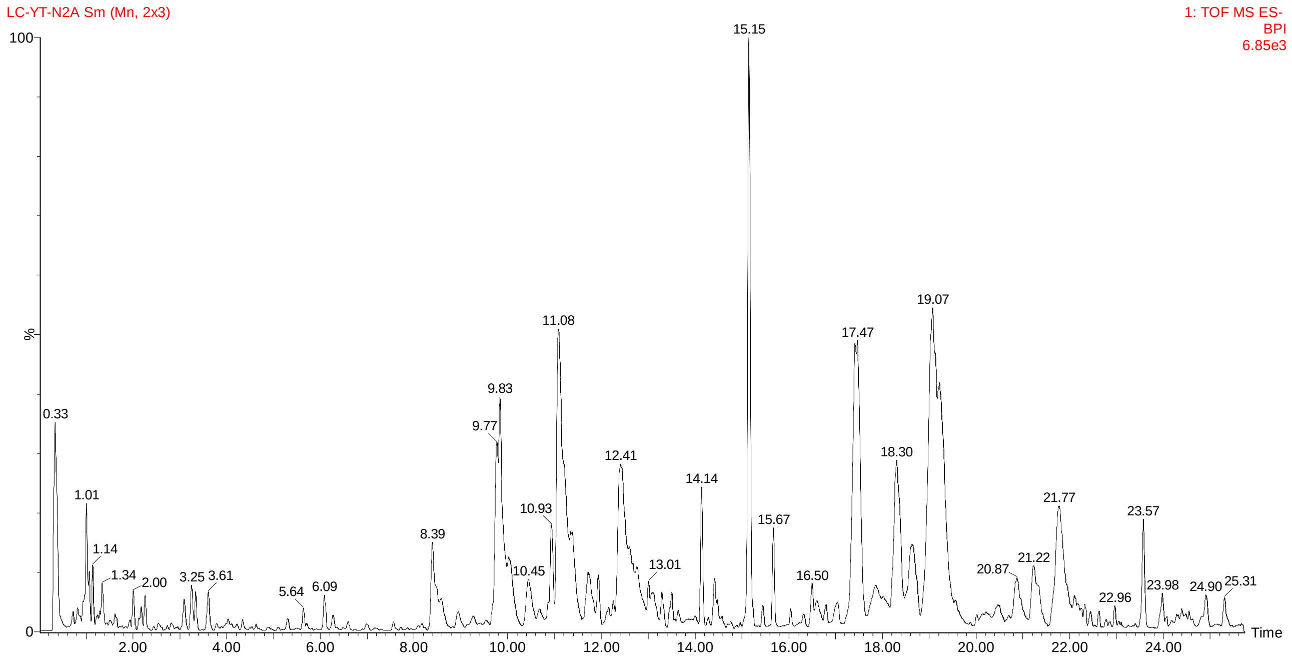

Figure S32. The positive BPI chromatogram of the sample B5

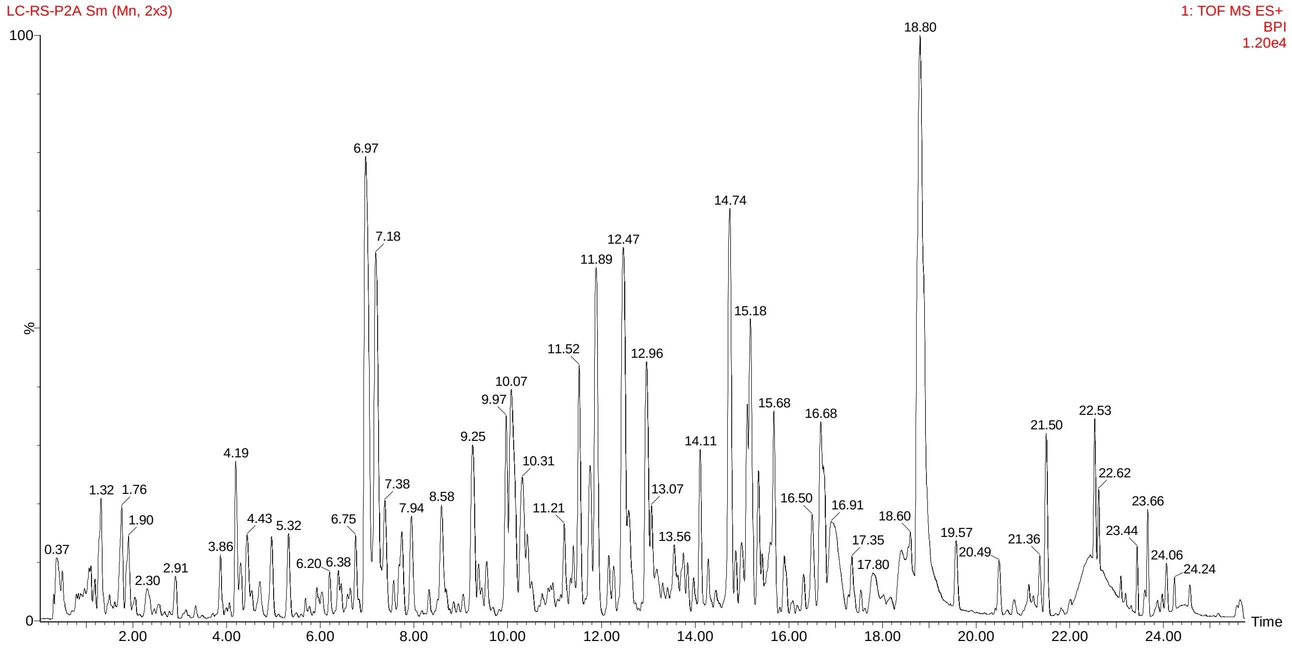

Figure S33. The negative BPI chromatogram of the sample B5

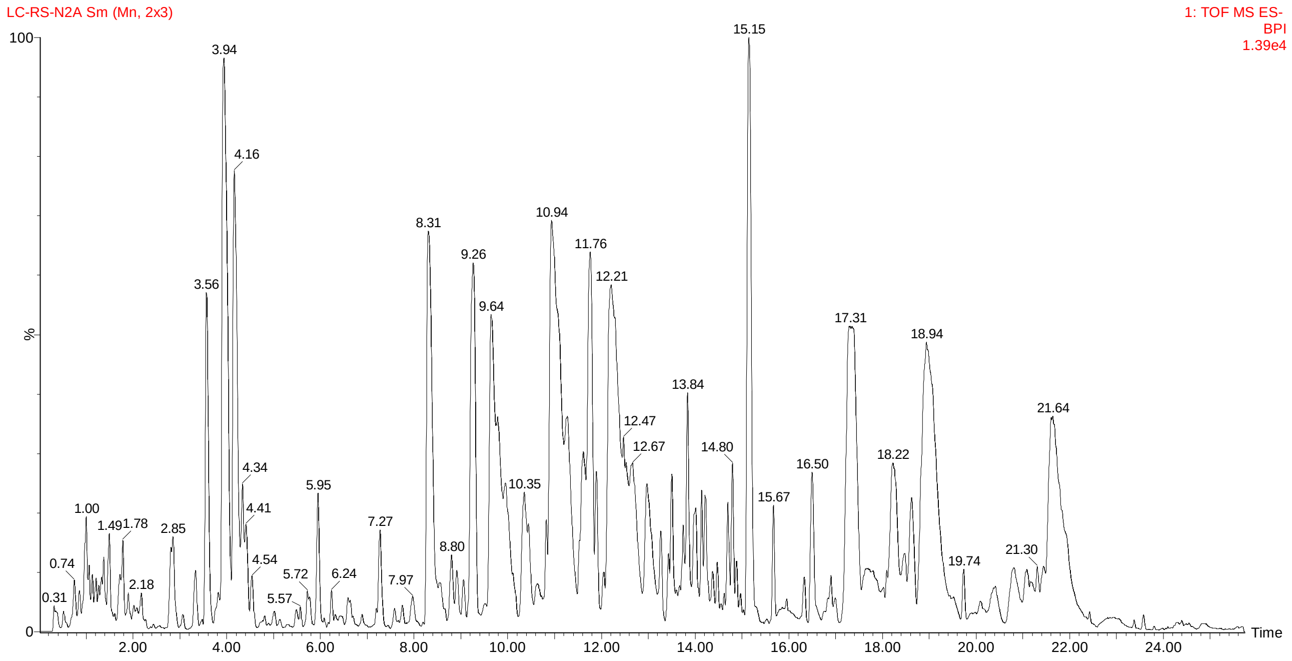

Figure S34. The positive BPI chromatogram of the sample B6

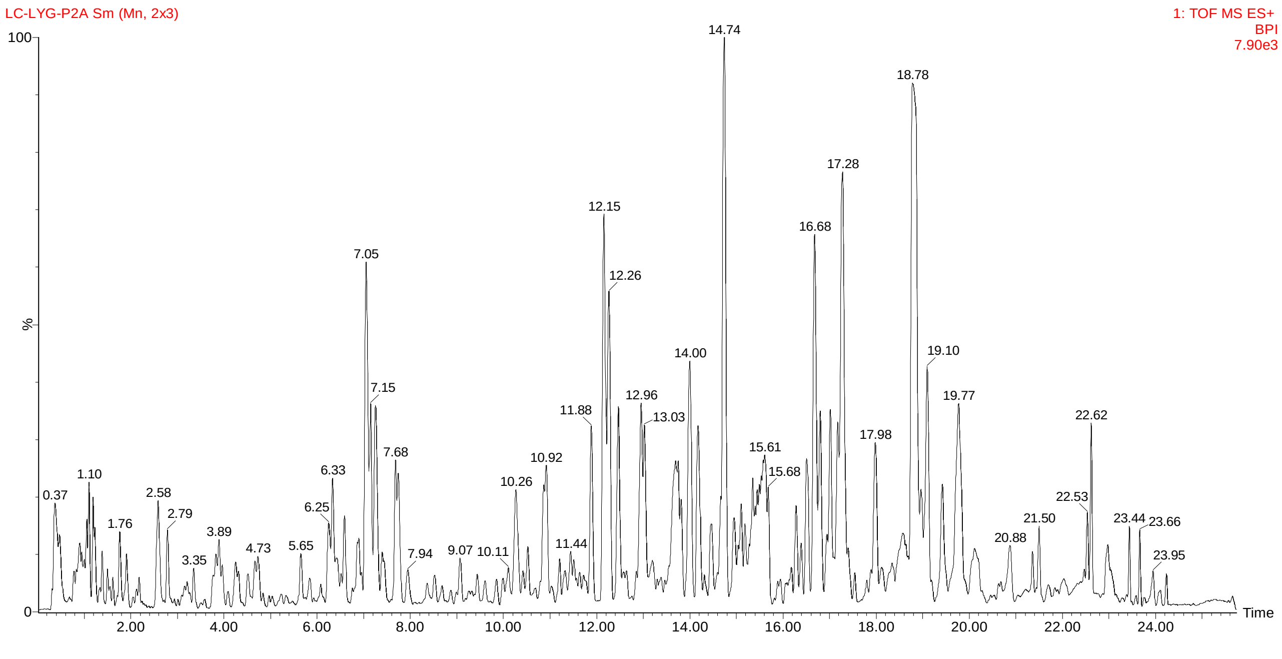

Figure S35. The negative BPI chromatogram of the sample B6

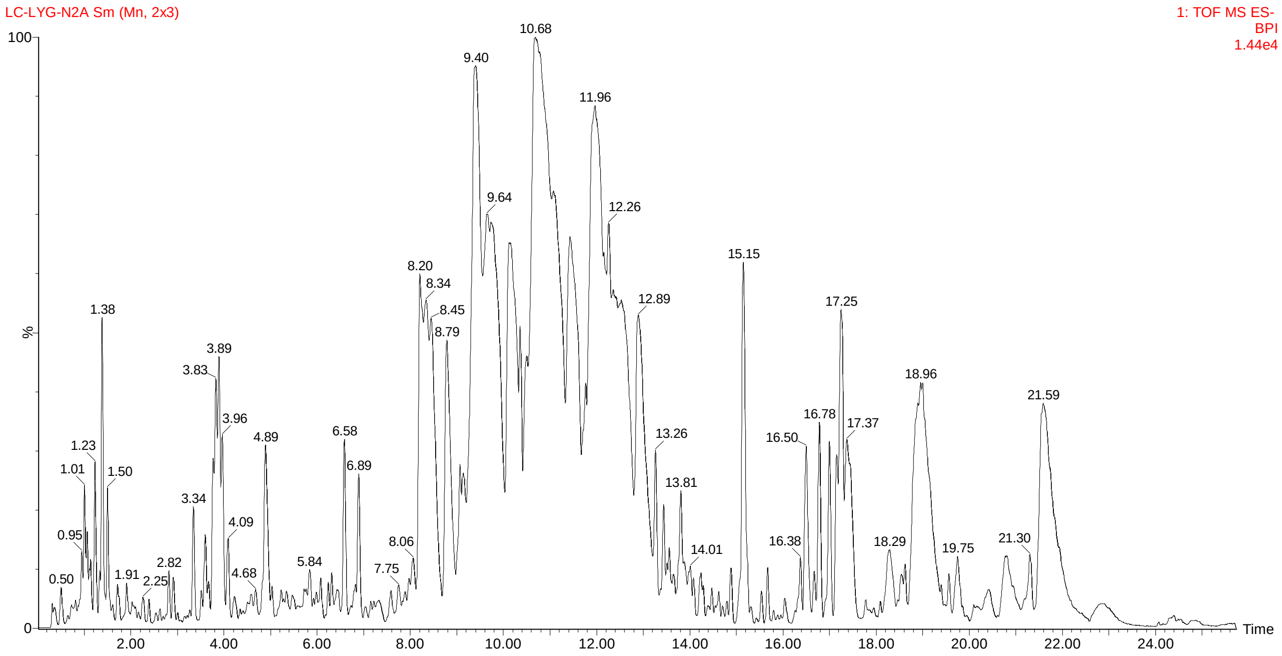

Figure S36. The positive BPI chromatogram of the sample B7

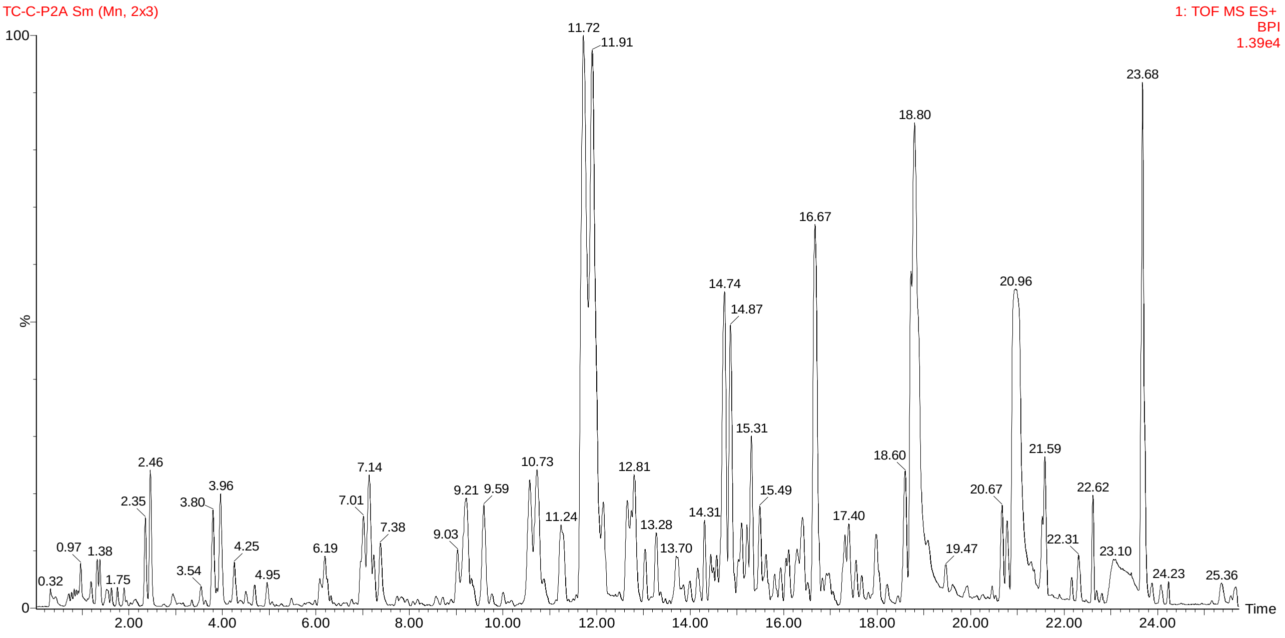

Figure S37. The negative BPI chromatogram of the sample B7

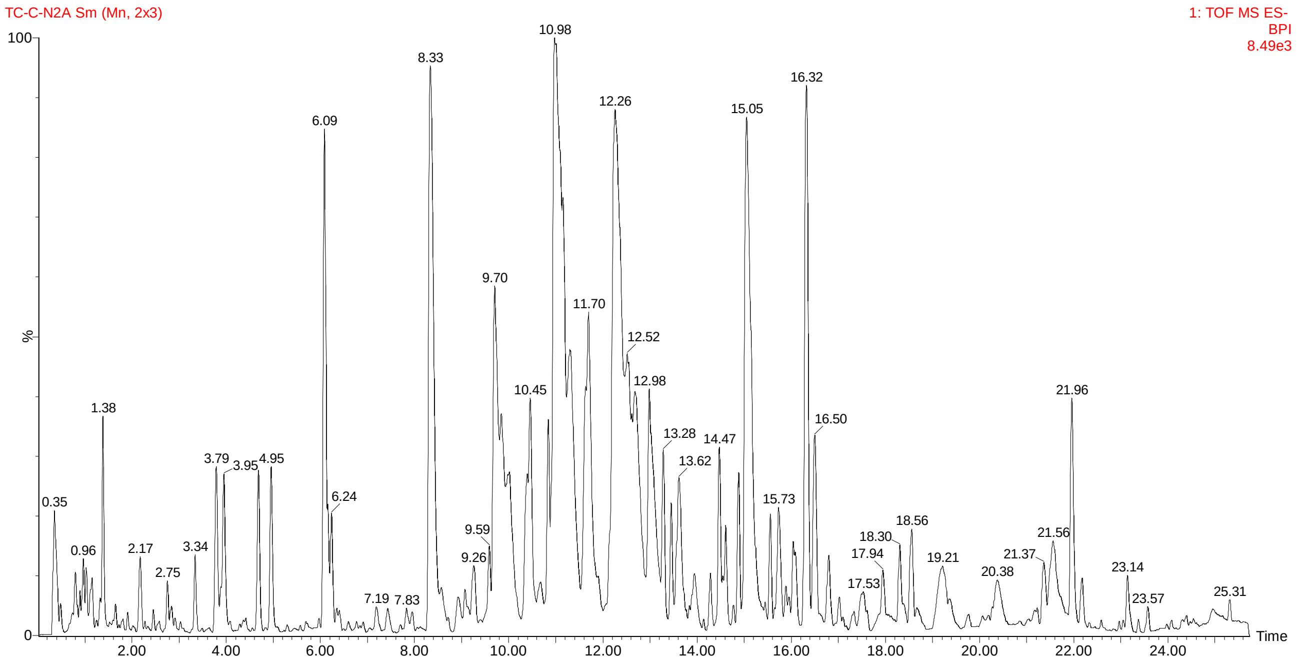

Figure S38. The positive BPI chromatogram of the sample B8

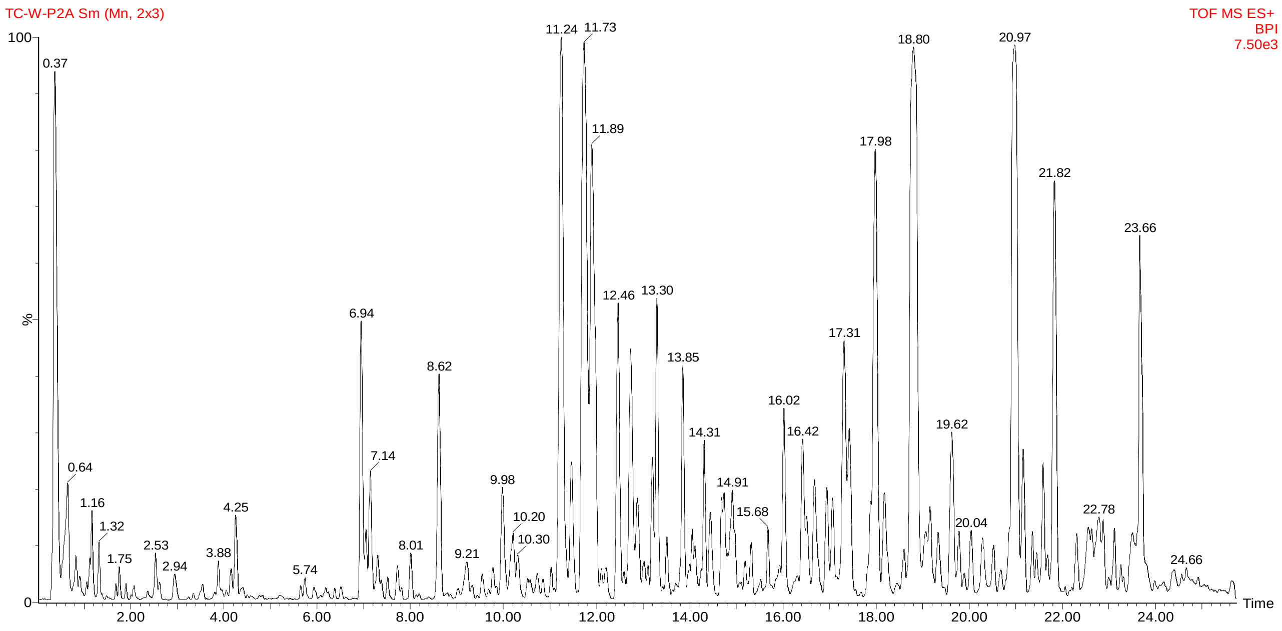

Figure S39. The negative BPI chromatogram of the sample B8

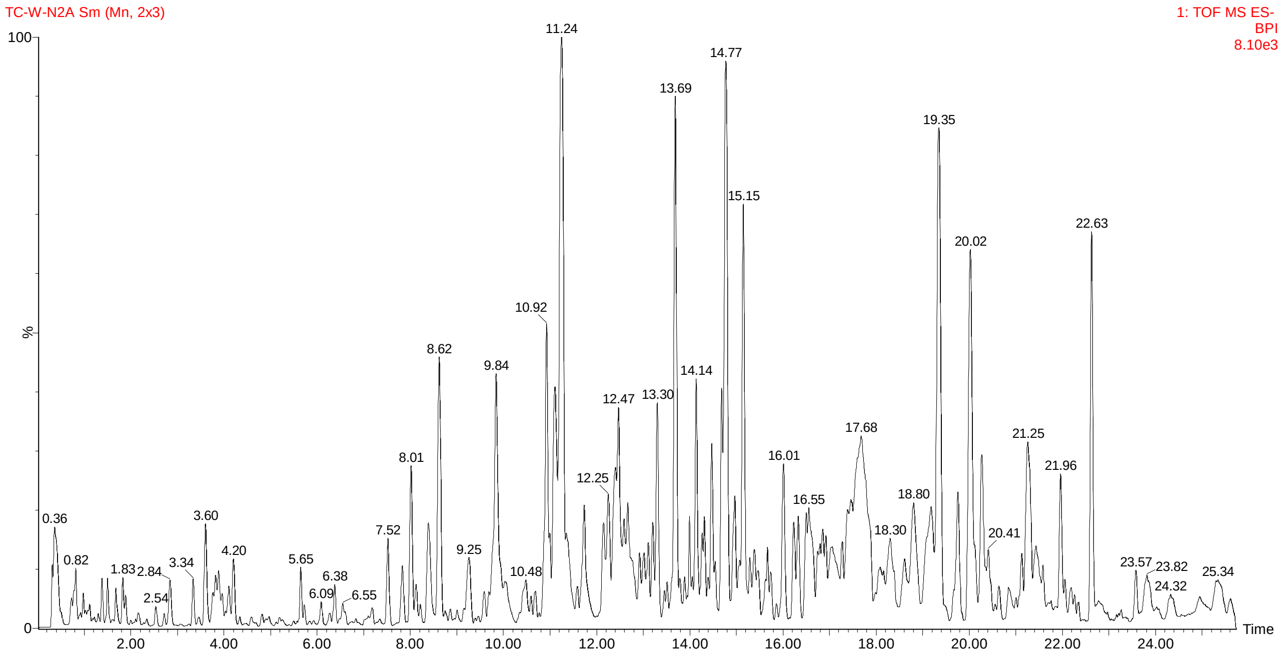

Figure S40. The positive BPI chromatogram of the sample B9

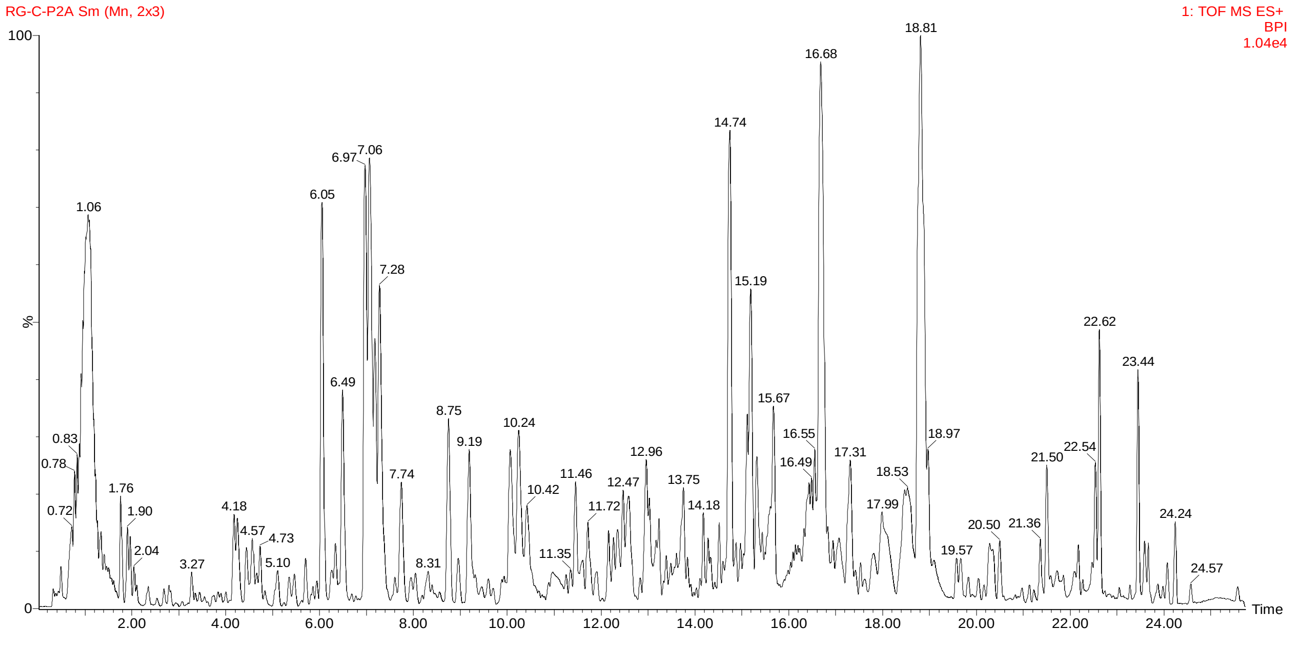

Figure S41. The negative BPI chromatogram of the sample B9

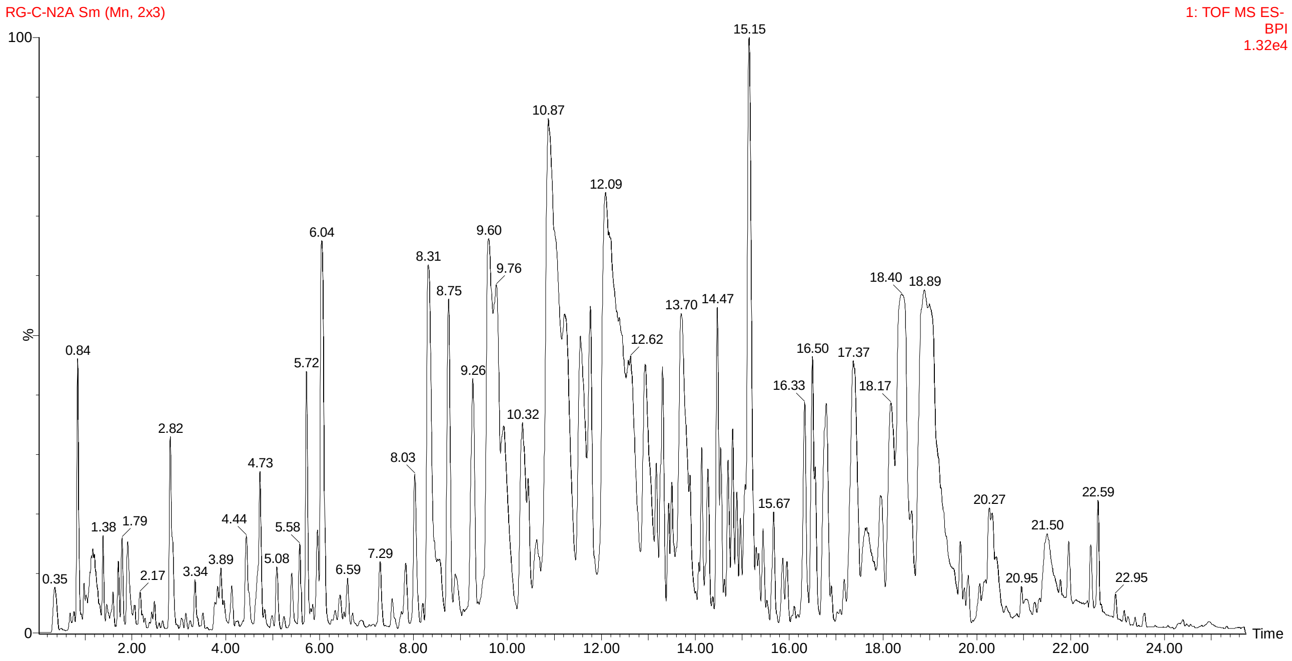

Figure S42. The positive BPI chromatogram of the sample B10

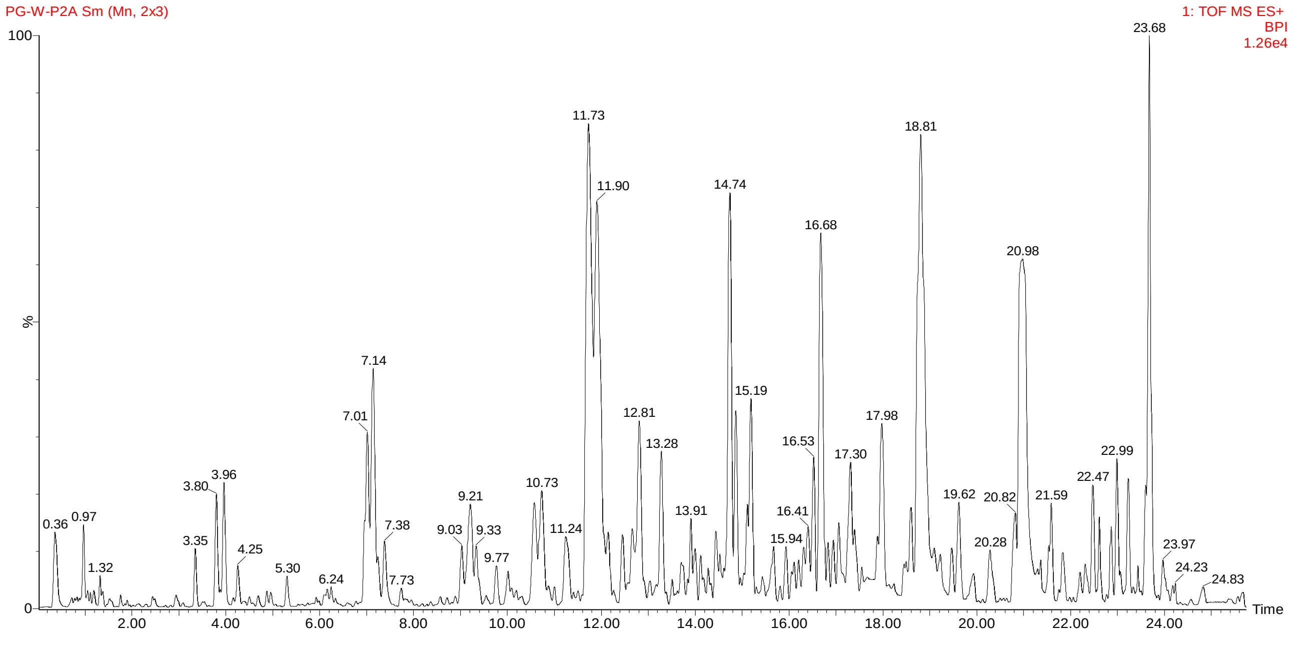

Figure S43. The negative BPI chromatogram of the sample B10

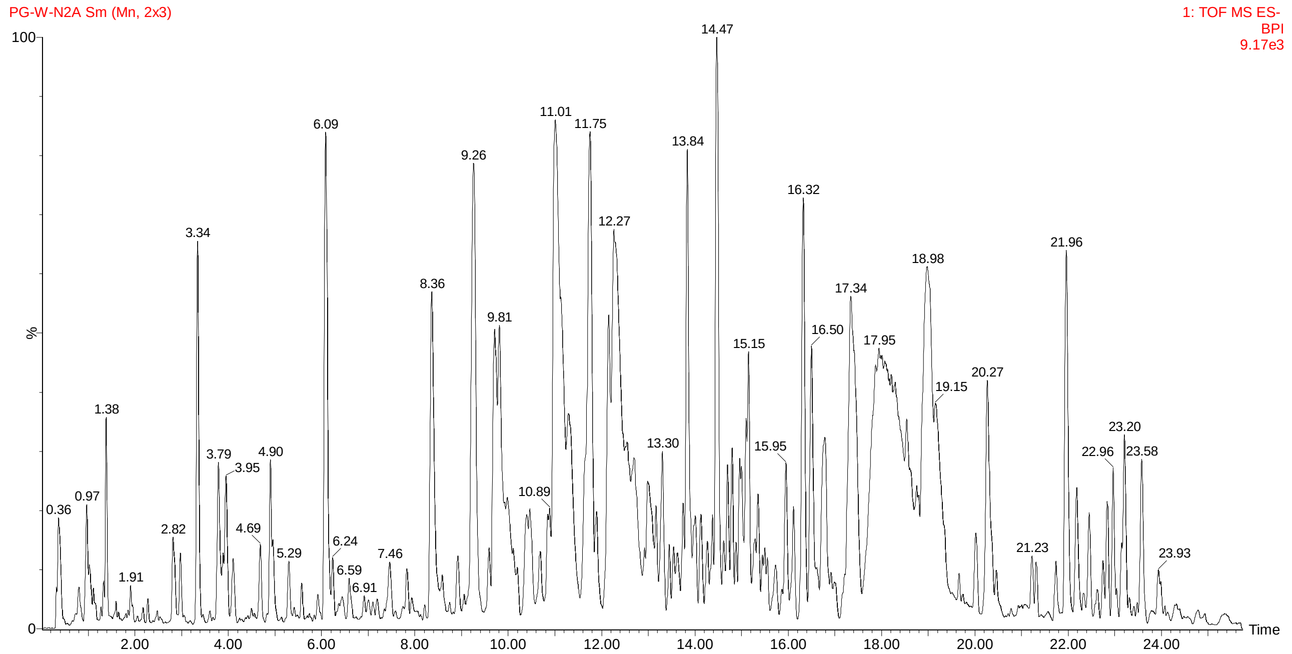

Figure S44. The positive BPI chromatogram of the sample B11

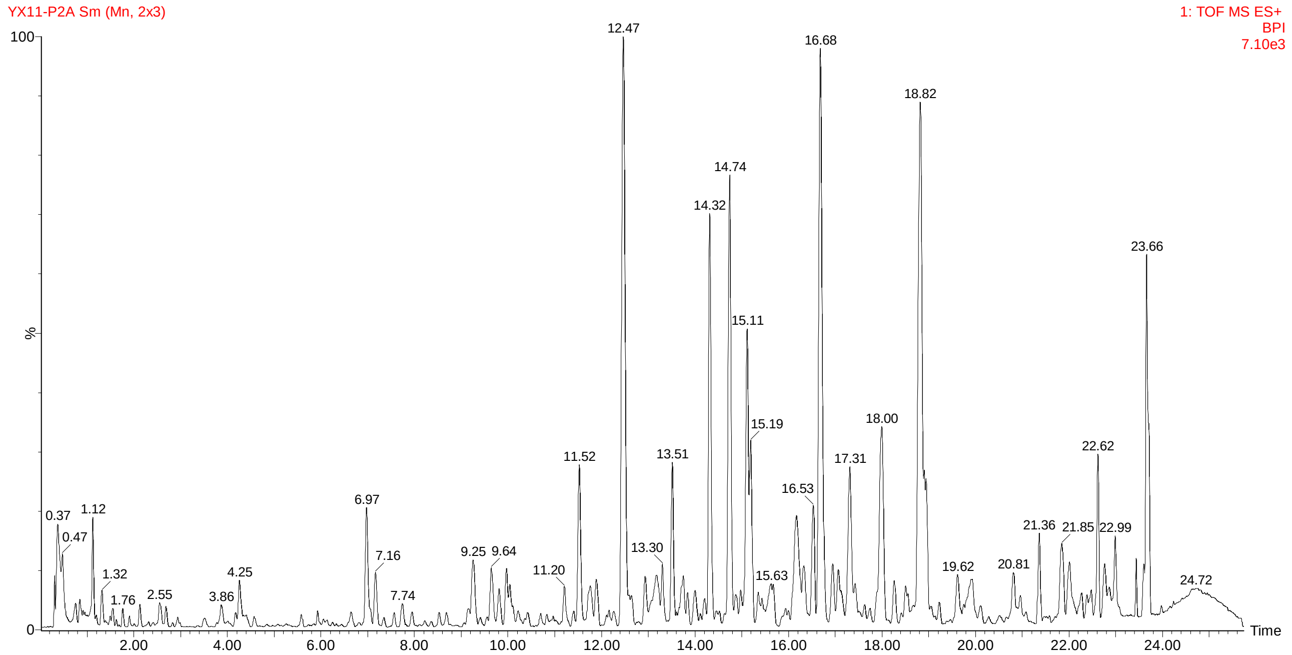

Figure S45. The negative BPI chromatogram of the sample B11

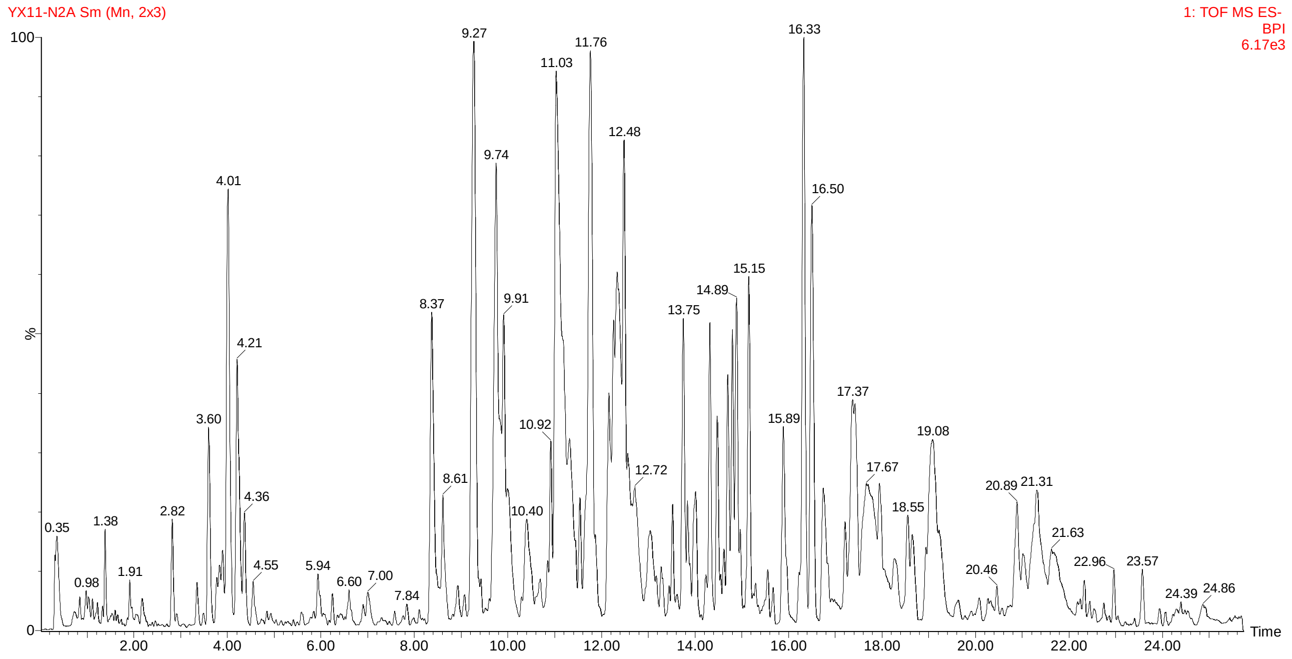

Figure S46. The positive BPI chromatogram of the sample B12

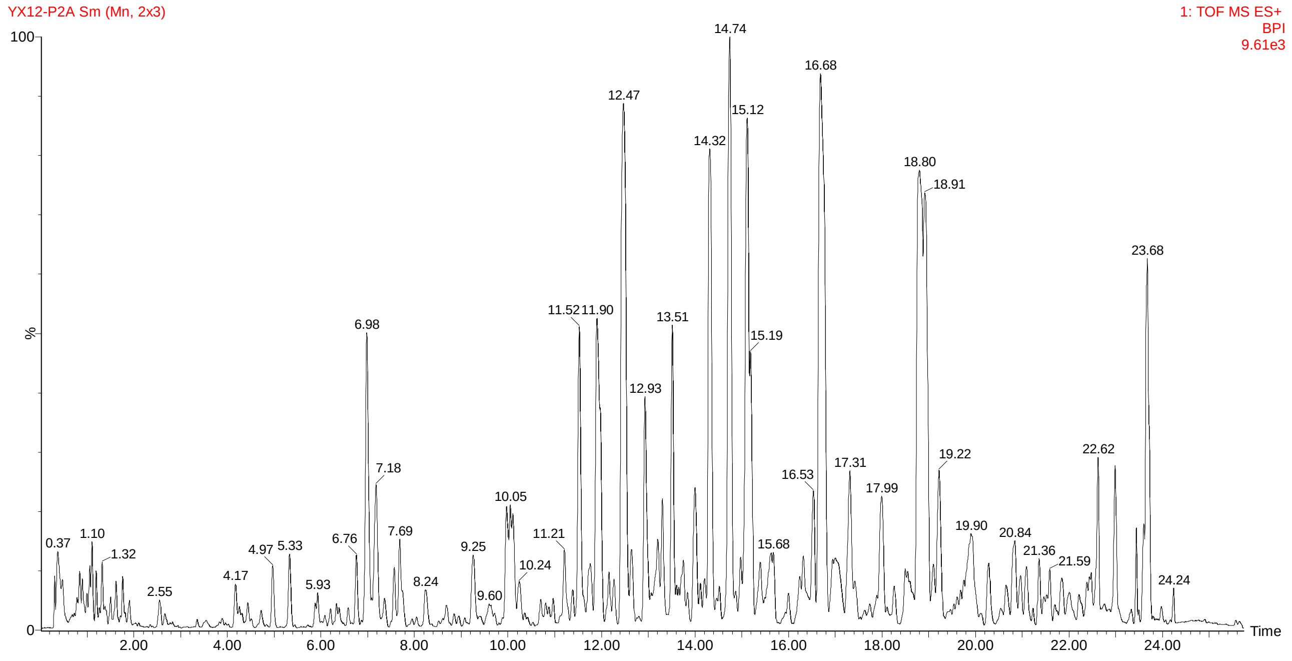

Figure S47. The negative BPI chromatogram of the sample B12

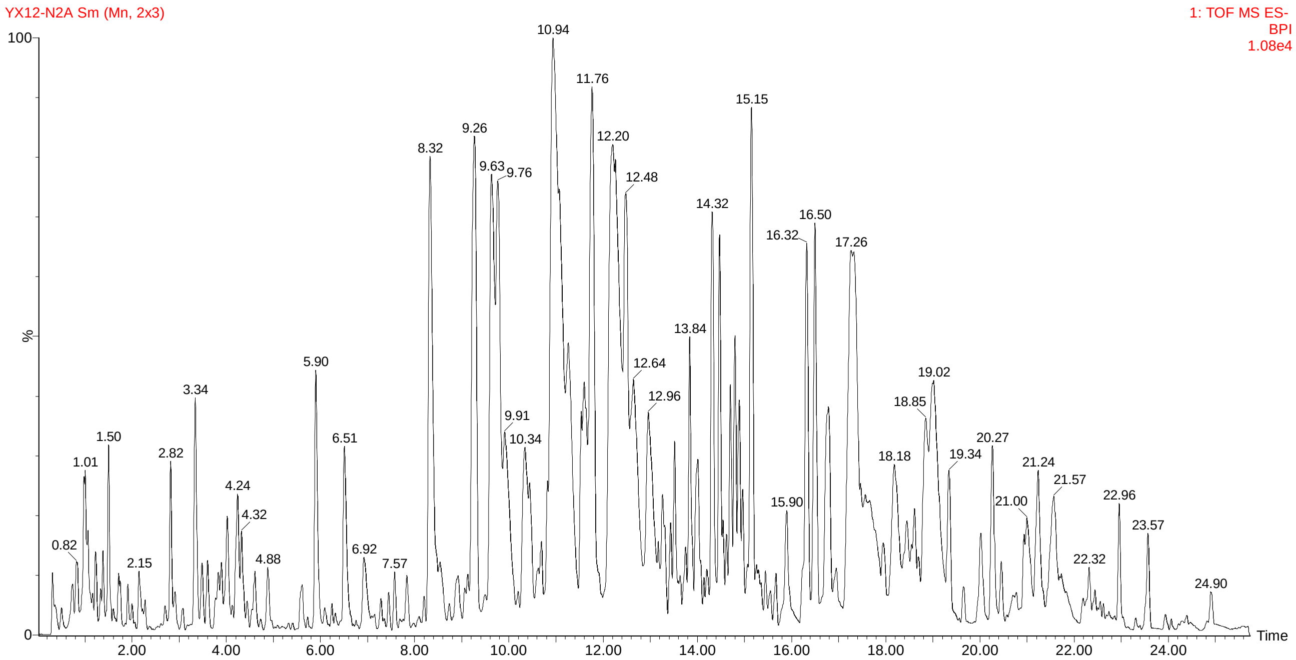

Figure S48. The OPLS-DA score plot in positive ion mode

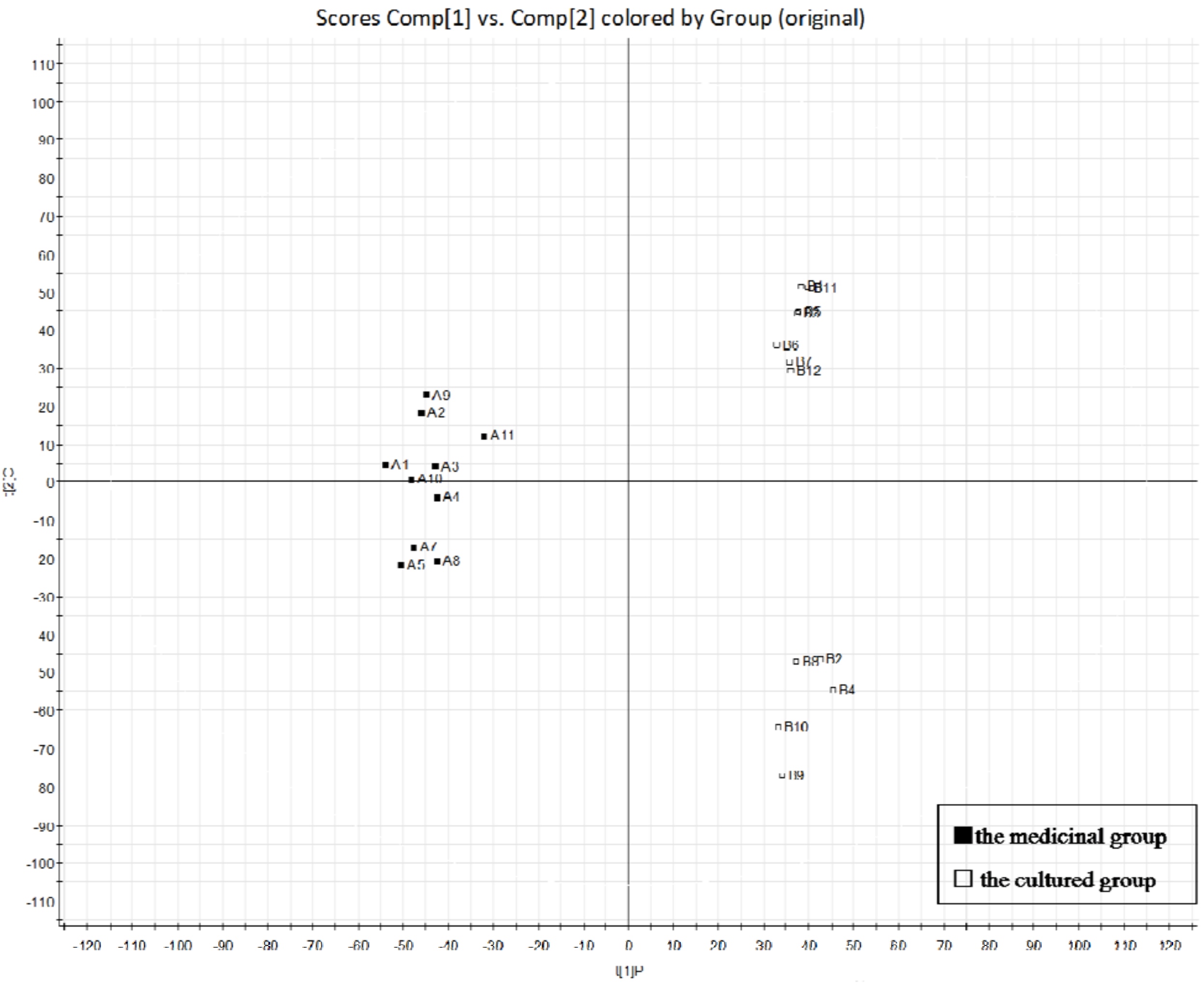

Figure S49. The ^{1}H-NMR spectrum of compound **1** in DMSO-d_6

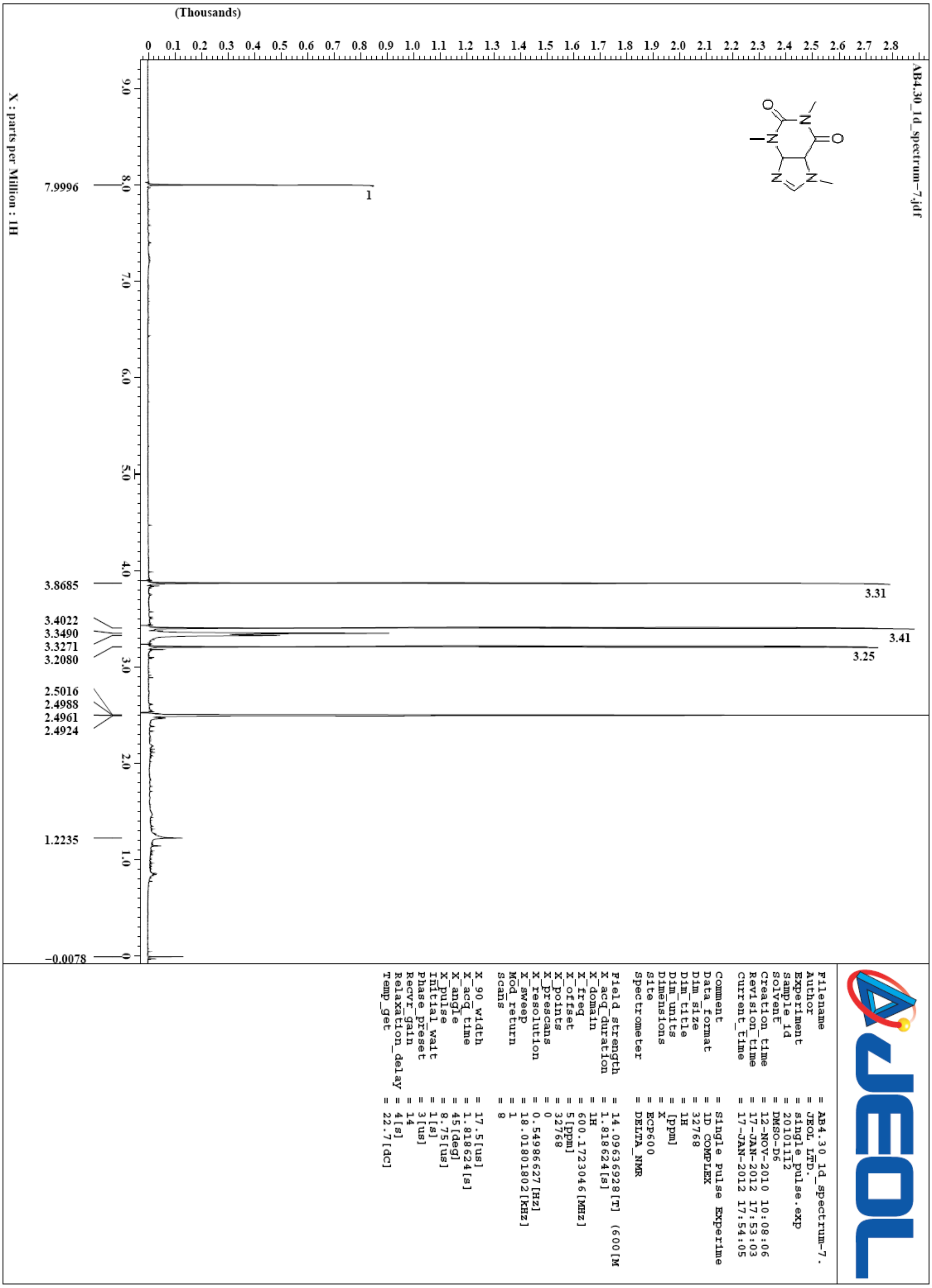

Figure S50. The ^{13}C-NMR spectrum of compound **1** in DMSO-d_6

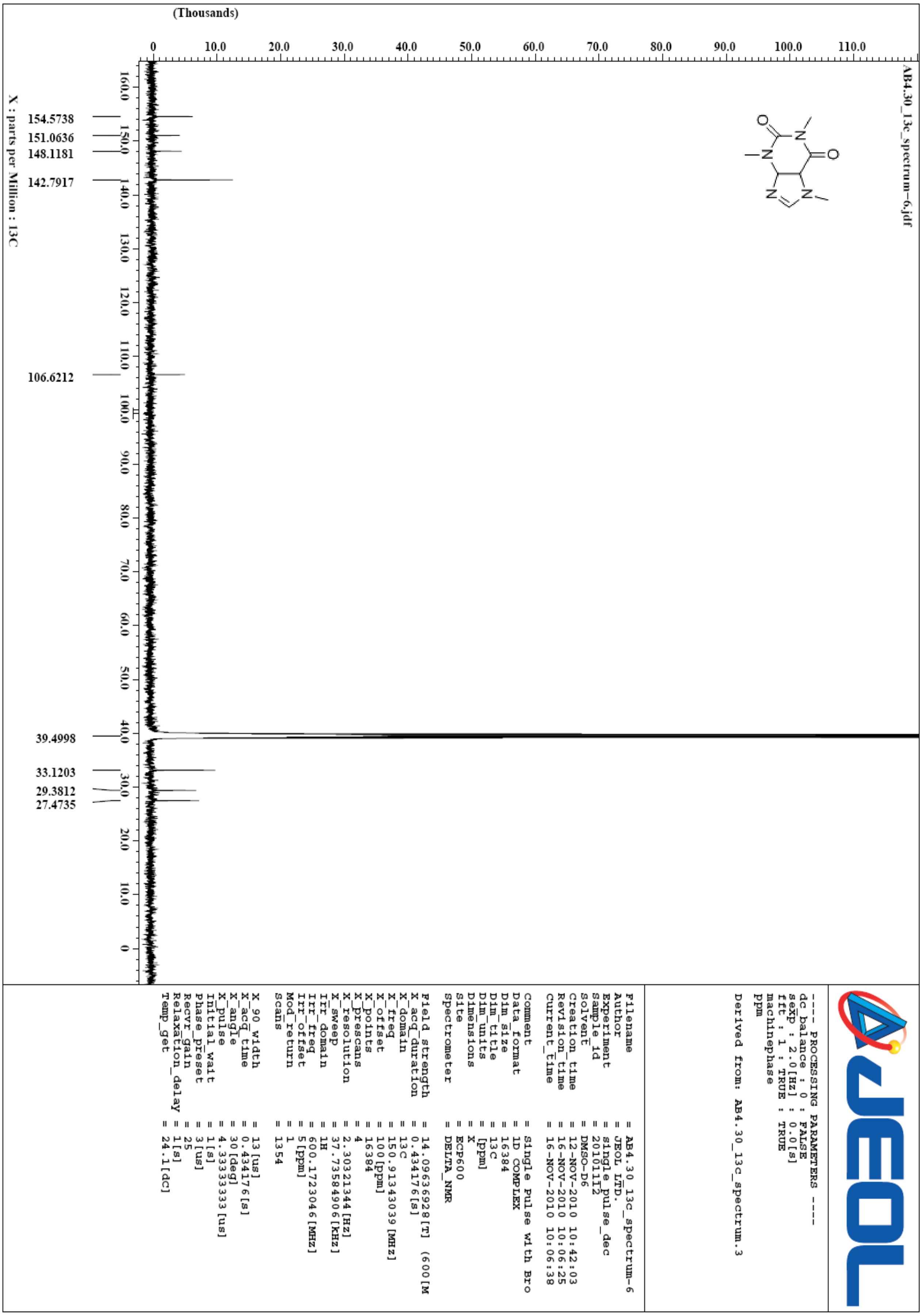

Figure S51. The HMQC spectrum of compound **1** in DMSO-d_6

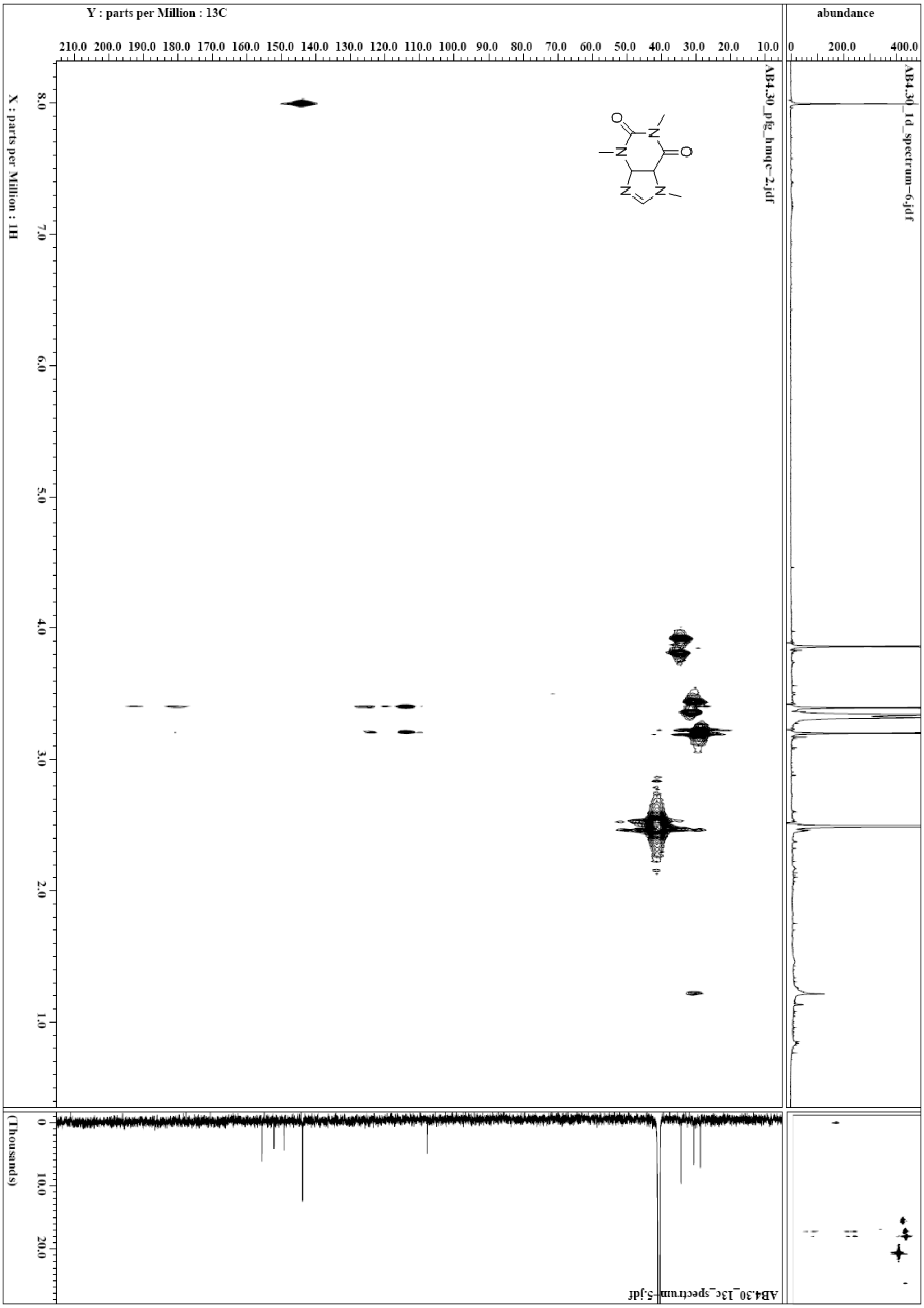

Figure S52. The HMBC spectrum of compound **1** in DMSO-d_6

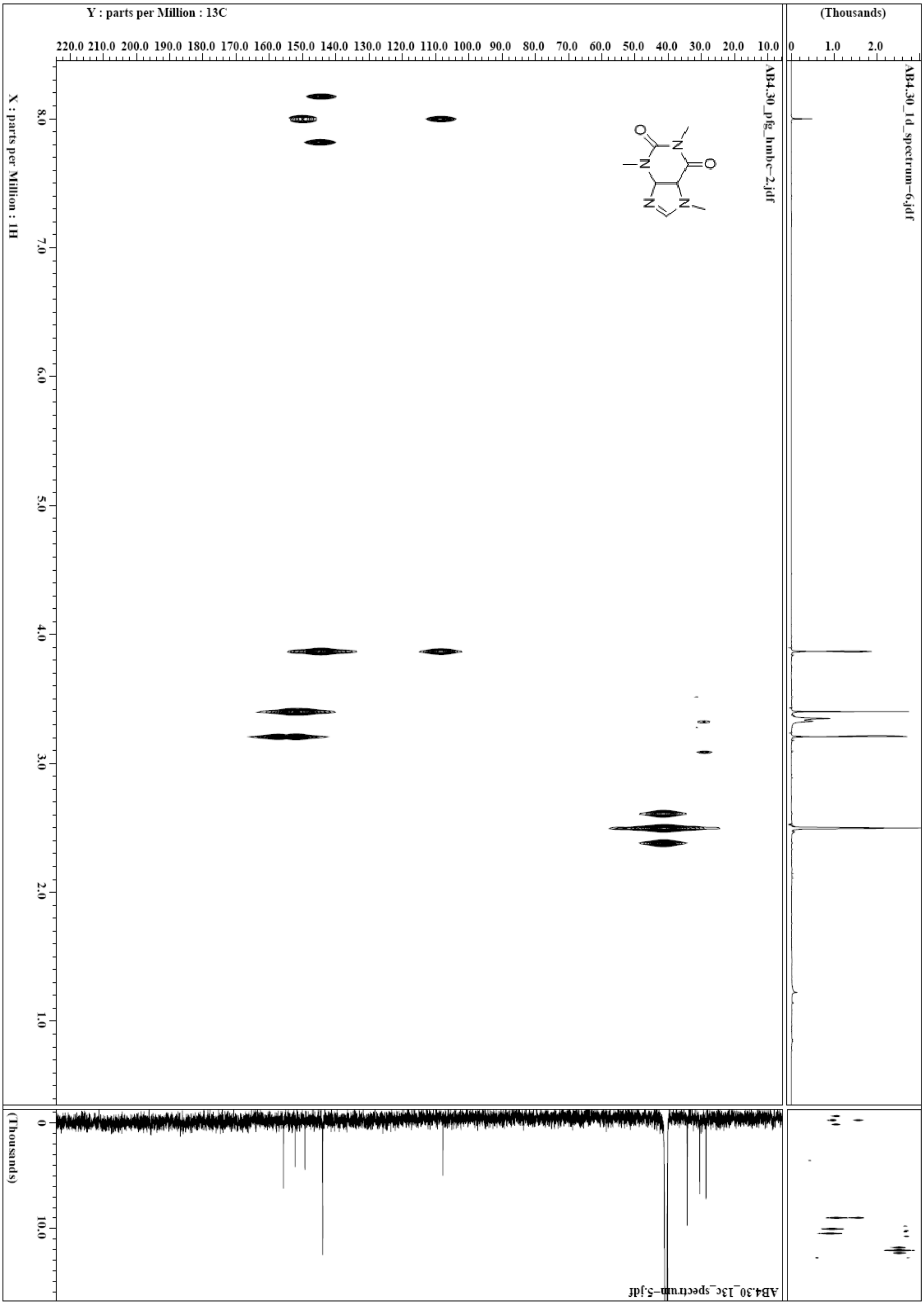

Supplement: Supplementary File 1: — PDF-Document (PDF, 1147 KB) [file marinedrugs-10-01180-s001.pdf]
